# Interaction of *Klebsiella pneumoniae* with tissue macrophages in a mouse infection model and ex-vivo pig organ perfusions: an exploratory investigation

**DOI:** 10.1016/S2666-5247(21)00195-6

**Published:** 2021-12

**Authors:** Joseph J Wanford, Ryan G Hames, David Carreno, Zydrune Jasiunaite, Wen Y Chung, Fabio Arena, Vincenzo Di Pilato, Kornelis Straatman, Kevin West, Robeena Farzand, Mariagrazia Pizza, Luisa Martinez-Pomares, Peter W Andrew, E Richard Moxon, Ashley R Dennison, Gian Maria Rossolini, Marco R Oggioni

**Affiliations:** a Department of Genetics and Genome Biology, University of Leicester, Leicester, UK; b Advanced Imaging Facility, University of Leicester, Leicester, UK; c Department of Respiratory Sciences, University of Leicester, Leicester, UK; d Department of Hepatobiliary and Pancreatic Surgery, University Hospitals of Leicester, Leicester, UK; e Department of Pathology, University Hospitals of Leicester, Leicester, UK; f Department of Clinical and Experimental Medicine, University of Foggia, Foggia, Italy; g IRCCS Don Carlo Gnocchi Foundation, Florence, Italy; h Department of Experimental and Clinical Medicine, University of Firenze, Firenze, Italy; i GlaxoSmithKline, Siena, Italy; j School of Life Sciences, Faculty of Medicine & Health Sciences, University of Nottingham, Nottingham, UK; k Department of Paediatrics, University of Oxford, Oxford, UK; l Clinical Microbiology and Virology Unit, Firenze Careggi University Hospital, Firenze, Italy; m Department of Farmacy and Biotechnology, University of Bologna, Bologna, Italy; n Department of Surgical Sciences and Integrated Diagnostics, University of Genoa, Genova, Italy

## Abstract

**Background:**

Hypervirulent *Klebsiella pneumoniae* (hv*Kp*) strains of capsule type K1 and K2 cause invasive infections associated with hepatic abscesses, which can be difficult to treat and are frequently associated with relapsing infections. Other *K pneumoniae* strains (non-hv*Kp*), including lineages that have acquired carbapenem resistance, do not manifest this pathology. In this work we aimed to test the hypothesis that within-macrophage replication is a key mechanism underpinning abscess formation in hv*Kp* infections.

**Methods:**

In this exploratory investigation, to study the pathophysiology of abscess formation, mice were intravenously infected with 10^6^ colony forming units (CFU) of either hv*Kp* isolates (six strains) or non-hv*Kp* isolates (seven strains). Intracellular bacterial replication and neutrophil influx in liver and spleen was quantified by fluorescence microscopy of sliced cryopreserved organs of mice collected 30 min, 6 h, and 24 h after infection with the aim to provide data of bacterial association to Kupffer cells in the liver and to the different tissue macrophages in the spleen. Microbiological and microscopy analysis of an ex-vivo model of pig liver and spleen infection were used to confirm within-macrophage replication. Pig organs were perfused with heparinised, autologous pig's blood and injected with 6·5 × 10^7^ CFU of hv*Kp* K2 sequence type 25 strain GMR151. Blood and tissue biopsies collected before infection and 30 min, 1 h, 2 h, 3 h, 4 h, and 5 h after infection were used to measure bacterial counts and to identify the subcellular localisation of bacteria by immunohistochemistry analysis.

**Findings:**

We show that hv*Kp* resisted phagocyte-mediated clearance and replicated in mouse liver macrophages to form clusters 6 h after infection, with a mean of 7·0 bacteria per Kupffer cell (SD 6·2); however, non-hv*Kp* were efficiently cleared (mean 1·5 bacteria per cell [SD 1·1]). Hv*Kp* infection promoted neutrophil recruitment to sites of infection, which in the liver resulted in histopathological signs of abscess formation as early as 24 h post-infection. Experiments in pig organs which share a high functional and anatomical resemblance to human organs, provided strong evidence for the propensity of hv*Kp* to replicate within the hepatic macrophages.

**Interpretation:**

These findings show subversion of innate immune processes in the liver by *K pneumoniae* and resistance to Kupffer cell mediated clearance as an explanation for the propensity of hv*Kp* strains to cause hepatic abscesses.

**Funding:**

University of Oxford and a Royal Society Wolfson grant funded biosafety facility.

## Introduction

*Klebsiella pneumoniae* invasive (pyogenic) liver abscess syndrome is an emerging, life-threatening disease that is very difficult to manage clinically and surgically.^1^ Since its initial description in Taiwan approximately 30 years ago,^2^ *K pneumoniae* has become the primary reported cause of pyogenic liver abscess in Asia, the USA, and Europe.^3^

*K pneumoniae* is a Gram-negative pathogen remarkably resistant to antibiotic therapy in the clinic. Within the species, two groups of strains pose distinct clinical problems; hypervirulent-hypermucoid lineages (hv*Kp*) cause severe systemic infection associated with liver abscesses and additional metastatic complications^4^ even in healthy populations, whereas the non-hypervirulent lineages (non-hv*Kp*) mostly cause urinary and lower respiratory tract infections and bacteraemia in compromised hosts. Resistance to antibiotics has spread in *K pneumoniae*, especially among non-hv*Kp*, and carbapenem-resistant strains are currently among the most challenging pathogens to treat.^5^, ^6^ Despite the burden of *K pneumoniae* disease, there is very limited understanding of its pathogenesis, particularly regarding tissue and cellular tropism.

Following entry into the blood, *K pneumoniae* are exposed to phagocyte-mediated clearance mechanisms in the liver and spleen.^7^ Both organs contain heterogeneous populations of macrophages^8^ but the roles of the various tissue macrophages in clearance of *K pneumoniae* are unknown, even if their role in the killing of encapsulated bacteria is well documented.^8^ Although *K pneumoniae* classically are considered to be extracellular pathogens, in-vitro studies showed *K pneumoniae* survival within macrophages^9^, ^10^ but the in-vivo significance of these observations was unclear. Consequently, we explored the interaction of *K pneumoniae* with tissue macrophages to determine whether there was evidence for in-vivo intracellular replication within macrophages, and if replication influenced the pathophysiology of liver abscess formation.

**Research in context**

**Evidence before this study**

Hypervirulent *Klebsiella pneumoniae* (hv*Kp*) strains are major clinical concern due to their ability to infect immunocompetent individuals, leading to severe systemic disease characterised by pyogenic tissue abscesses. Non-hv*Kp* strains mainly infect immunocompromised individuals and are characterised by high levels of drug resistance. Optimisation of therapeutic interventions against *K pneumoniae* infection would benefit from an understanding of the innate immune response to microbial challenge, of which macrophage-driven clearance is a key facet. We, therefore, searched PubMed and Google Scholar for studies published between Jan 1, 1990, and Jan 1, 2021, containing the terms “*K. pneumoniae*”, “intracellular replication”, and “macrophages”. We included only articles in English. We identified two studies that showed the propensity for some strains of *K pneumoniae* to persist, and in some cases replicate, within immortalised alveolar macrophage cell lines. Importantly, we found no evidence for analysis of *K pneumoniae* interaction with tissue macrophages in the liver or spleen, two major sites of bacterial metastasis during infection.

**Added value of this study**

Our study is the first to investigate the tropism of clinical isolates of *K pneumoniae* in the liver and spleen tissue in vivo in a mouse model and in a novel ex-vivo pig model. Our data showed that hv*Kp* strains persisted for at least 6 h within tissue macrophages in both the mouse spleen and liver, whereas non-hv*Kp* did not. Macrophage infection by hv*Kp* strains proceeded localised influx of neutrophils, that developed into micro-abscesses, which were unable to clear infecting bacteria. Non-hv*Kp* were efficiently cleared in the tissue and showed no hallmarks of abscess formation. Using a novel model of ex-vivo pig liver and spleen perfusion we showed the propensity of hv*Kp* to replicate intracellularly in macrophages and provided a preclinical model of *K pneumoniae* infection in a natural host that will facilitate testing of therapeutic approaches, in a model directly translational to humans.

**Implications of all the available evidence**

Pyogenic liver abscesses are an increasing health-care emergency posing substantial challenges for medical and surgical treatment. Our insight into the pathophysiology of disease provides a strong imperative to confirm these observations in humans, with the view to reconsider treatment regimens for hv*Kp* infection with a focus on using antimicrobials that have a higher propensity to accumulate within host cells. Further, our findings open the door to the development of host-directed therapeutics enhancing macrophage-mediated killing, a strategy that might prove effective in the face of emerging pan-drug resistance in this clinically important human pathogen.

## Methods

### Study design

This work was an exploratory study analysing the tropism of both hypervirulent and classical *K pneumoniae* in mouse infection models, in-vitro cell culture models, and an ex-vivo pig organ perfusion model.

### Bacterial strains and culture conditions

We used 13 *K pneumoniae* strains of clinical origin, encompassing five capsular serotypes, including three hv*Kp* strains of serotype K1 (NTUH-K2044, RM1628, SGH10), three of serotype K2 (GMR151, HMV-1, HMV-2), three non-hv*Kp* carbapenem-resistant strains of serotype KL17 (DG5544, KPC58, KPC284), one of serotype KL103 (HS11286), and three of serotype KL107 (KPC157, KKBO-1, KK207-1; appendix p 2). Full details of bacterial growth conditions can be found in the appendix (p 22).

### Mouse intravenous sepsis model

All animals used in this study were handled in accordance with the UK home office project licence P7B01C07A. Procedures and experiments were approved by the local University of Leicester Research Ethics committee. Groups of five mice were randomly allocated to cages using a random number generator and maintained in the Pre-clinical Research Facility at the University of Leicester with a 0700–1900 h lighting cycle, under standard practices for husbandry. For the sepsis model, female 1 Swiss mice, aged 6–8 weeks, between 20 g and 25 g, bred in house were intravenously infected via the lateral tail vein with 10^6^ colony forming units (CFU) of *K pneumoniae* in 100 μL phosphate buffered saline (MP Biomedicals, Irvine, CA, USA) between 0900 h and 1100 h.^11^ In all cases, the order in which the mice were infected was the same as the order in which they were humanely killed at the endpoint. Infections and sample processing were not masked. A single replicate screening experiment was done, in which individual mice were challenged with all 13 individual *K pneumoniae* strains (appendix p 2), before analysing bacterial titres and distribution in blood, liver, and spleen by CFU plating and microscopy at 6 h after infection. A co-infection with strains of different capsular types (strains K1-RM1628 and K2-GMR151, dose 10^6^ CFU per mouse, three mice per group, 6 h liver and spleen sampling) was performed to test for multiple uptake of bacteria into tissue macrophages. A time course experiment was done with groups of five mice infected with representative K1 (NTUH-K2044) and K17 (KPC58) strains. Mice were monitored for signs of disease (hunching, movement, and piloerection) regularly throughout infection. At 30 min, 6 h, 24 h, and 48 h after infection, blood was removed by cardiac puncture under terminal anaesthesia, and placed into microcentrifuge tubes containing 50 units of heparin (Sigma, Gillingham, UK). Liver and spleen samples were removed following cardiac puncture, both were cut into two pieces coronally, and either homogenised in brain heart infusion broth (Oxoid, Basingstoke, UK) with 10% v/v glycerol for enumeration of CFU, or flash frozen in Optimum Cutting Matrix (Fisher Scientific, Waltham, MA, USA) using 2-methyl butane (Sigma) and dry ice for microscopy analysis. Serum was collected from naive mice in the same colony to determine anti-*K pneumoniae* IgG titres of the animals and was prepared by allowing whole blood to clot on ice for 10 min, before centrifugation at 400 × g for 30 min. The serum was stored at −80°C.

### Ex-vivo perfusion and infection of pig liver and spleen

Spleens, livers, and blood from three food chain-sourced Large White pigs were retrieved from Joseph Morris Abattoir (South Kilworth, UK). The organs were cannulated in situ via the splenic artery, hepatic artery, and portal vein before being transported to the Leicester General Hospital (UK), where organs were perfused with 2 L of heparinised, autologous pig's blood.^12^, ^13^ Throughout the perfusion, maintenance of physiological blood gas parameters was monitored using a Radiometer ABL90 series (Radiometer UK, Sussex, England). For infections, three independent perfusion circuits (consisting of one liver and one spleen in each experiment) were injected with 6·5 × 10^7^ CFU of K2 sequence type (ST) 25 strain GMR151. This strain was selected because K2 ST25 strains are a known cause of infections in pigs.^14^ Serial blood and tissue biopsies were collected before infection and 30 min, 1 h, 2 h, 3 h, 4 h, and 5h after infection. Blood and homogenised tissue biopsies were serially diluted to determine bacterial counts or flash frozen in optimal cutting temperature compound (CellPath, Newton, UK) for immunohistochemistry analysis. Additional pig blood samples were taken for investigation of anti-*Klebsiella* serum bactericidal activity.

### Cell culture, macrophage infection, and primary neutrophil isolation

J774A.1 macrophages (American Type Culture Collection TIB67) were maintained in Dulbecco's Modified Eagle Medium (Gibco, Waltham, MA, USA) plus 10% Fetal Calf Serum (Gibco, Waltham, MA, USA). Primary neutrophils were obtained from CD1 mouse blood and isolated as described earlier, using gradient centrifugation. Cells were confirmed as neutrophils before infection by positive immunostaining following paraformaldehyde fixation with an anti-Ly-6G monoclonal antibody. Detailed protocols for culture and infection of both cell types are given in the appendix (pp 23–24). Macrophages were infected (detailed protocol in the appendix [pp 23–24]) with K1 strains NTUH-K2044 and SGH10, the K107 strain KPC157, and the K17 strain KPC58 at a multiplicity of infection of ten. Gentamicin treatment at 300 μg/ml for 1 h was added to macrophage cultures to kill extracellular bacteria (appendix p 23), after further incubation for 1 h or 4 h cells were either lysed to enumerate intracellular organisms or fixed in 4% paraformaldehyde for immunofluorescence staining. The gentamycin protection assay used gentamycin 300 μg/ml for 1 h on tissue homogenates with a cell lysis step in 0·1% w/v saponin either before or after the antibiotic treatment (appendix pp 23–24). Primary neutrophils in Hanks' balanced salt solution with Ca^2+^ and Mg^2+^ were inoculated with K1 strain NTUH-K2044, K2 strain GMR151, K107 strain KPC157, or K17 strain KPC58 at a multiplicity of infection of 10, incubated for 2 h at 37°C under rotation before cell lysis and enumeration of intracellular organisms.

### Immunohistochemistry and fluorescence microscopy analysis

Frozen mouse and pig organ sections (10 μm) were mounted onto microscope slides, following sectioning with a Leica cryostat (CM1850UV, Leica Biosystems, Wetzlar, Germany). Fluorescence labelling of tissue sections was done as before,^11^ and all antibodies and reagents used are shown in the appendix (p 3). Histological analysis of tissue sections was done by light microscopy following hematoxylin (Mayer's, Sigma, Gillingham, UK) and eosin (CellPath, Newtown, UK) staining at the University of Leicester Histology Service. For analysis of bacterial distribution in tissues, immunostained tissue sections were imaged on a Vectra Polaris digital pathology system (Perkin Elmer, Akoya Biosciences, Marlborough, MA, USA; hereafter referred to as a slide scanner). Z-stack images and single optical sections of tissue samples stained with surface markers for Kupffer cells (F4/80), red pulp macrophages (F4/80), metalophillic macrophages (CD169), and marginal zone macrophages (MARCO^+^) were taken with an FV1000 Olympus Confocal laser scanning microscope (Olympus Life Science Solutions, Tokyo, Japan). Z-stack images were used to delineate subcellular localisation of bacteria and single optical sections to count the size of cell-associated bacterial clusters. Immunostained primary neutrophils and macrophage infections were imaged using the confocal system. Confocal microscopy images were visualised in Fiji v2.0.0-rc 69/1.52p and Imaris 3D V9.4 (Bitplane, Switzerland) reconstruction software was used for 3D visualisation of Z-stacks (appendix pp 22–23).

### Whole-cell ELISA

Mouse and pig sera were analysed for the presence of anti-*K pneumoniae* antibodies to clarify the role of antibodies in the observed clearance of bacteria in both the in-vivo and ex-vivo model. Briefly, plates coated with *K pneumoniae* K2 strain GMR151 were probed with serum samples, before detection with an anti-mouse (Sigma) or anti-pig (Sigma) IgG alkaline phosphatase conjugated secondary antibody. Full details of the experimental procedure are given in the appendix (pp 24–25).

### Statistical analysis

Bacterial titres (CFUs) in the tissue are the experimental measurement with the highest amount of variation between animals. Our pilot data indicate that CFUs in our model do not follow a normal distribution; therefore, we analysed the natural log-transformed values. Using power calculations described by Lenth^15^ for two-way ANOVA for analysis of differences in log-transformed CFU between hv*Kp* and carbapenem-resistant *K pneumoniae* in multiple organs, computing sample size for 80% power, and defining statistical significance as a p value of less than 0·05, with an expected effect size of 1·5, we required a group size of five mice. For comparison of two groups in microscopy analysis of infected tissue sections that followed a normal distribution we used a *t* test. For comparison of multiple groups, a one-way ANOVA was done, with Tukey's correction for multiple comparisons. p values were considered significant if they were less than 0·05. The statistical analysis pipeline and p values for all comparisons are shown in the appendix (p 6).

### Role of the funding source

The funder of the study played no role in study design, data collection, data analysis, data interpretation, or writing of the report.

## Results

**Figure 1:**
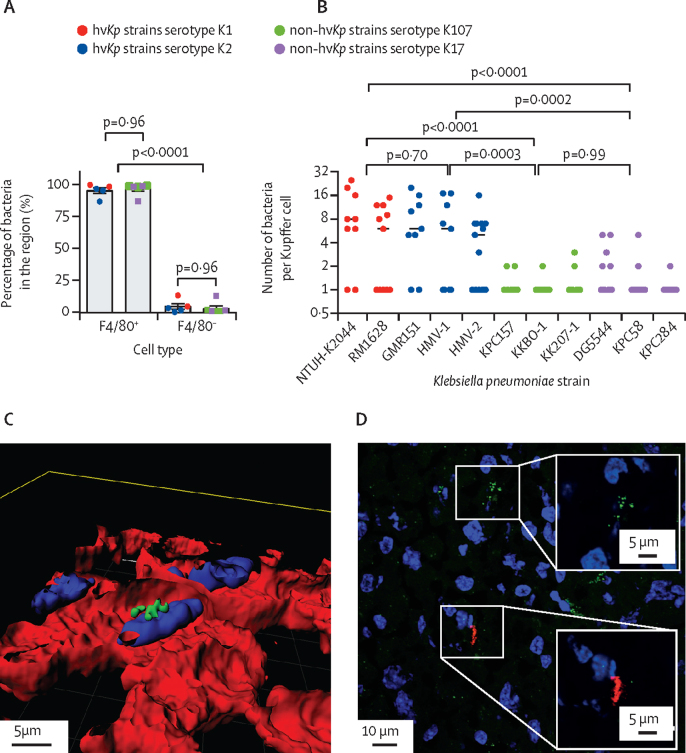
Tissue tropism and intracellular localisation of *Klebsiella pneumoniae* in mouse livers 6 h after intravenous infection (A) Quantitative distribution of *K pneumoniae* in F4/80^+^ and F4/80^−^ cells in the mouse liver. Data are representative of two entire tissue sections of approximately 2 × 2 cm per mouse. (B) Size of bacterial clusters associated with Kupffer cells at 6 h after infection. 30 random fields of view were analysed with a 60 × magnification with the Olympus confocal microscope. Circles represent individual bacterial clusters. (C) 3D reconstruction using Imaris 3D V9.4 of a K1 foci of infection Z-stack acquired on the confocal microscope showing the top of the cell layer, and a single cut into the cell. DAPI stain indicated in blue, F4/80 in red, and K1 ST23 in green. (D) K1 and K2 co-infected tissue section stained with purified IgG raised against K1 (green) capsule, or K2 (red) indicating two monochrome bacterial foci. A higher magnification of each monochrome focus is shown in insets. Lower magnification co-localisation images for all serotypes, in addition to confocal Z-stacks are shown in the appendix (pp 7–8). DAPI=4′,6-diamidino-2-phenylindole. hv*Kp*=hypervirulent *K pneumoniae*.

To test whether hv*Kp* and non-hv*Kp* strains behaved differently in a mouse model of invasive infection, individual mice were intravenously infected with one of a panel of six hv*Kp* and seven non-hv*Kp* strains (13 total) representative of circulating lineages in humans that expressed different capsular types (appendix p 2). In the liver at 6 h after infection, almost all bacteria (86–99%), regardless of hypervirulence, co-localised with the F4/80^+^ Kupffer cells (figure 1A). Representative co-localisation images are shown for K1-ST23 strain NTUH-K2044 (appendix pp 7–8). Similar co-localisation images were obtained for K2-ST25 strain GMR151, K17-ST101 strain KPC58, and KL107-ST512 strain KPC157 (appendix pp 7–8). Higher resolution confocal microscopy allowed quantification of the number of bacteria associated with each Kupffer cell. These data showed that the number of hv*Kp* associated with a single Kupffer cell (mean 7·0 bacteria per cell [SD 6·2]) was greater than for any non-hv*Kp* strain (1·5 bacteria per cell [1·1]; figure 1B). Bacteraemia levels were not consistent within the hv*Kp* group, with K2 isolates showing higher titres (appendix pp 7–8). For all strains, confocal images showed localisation within the Kupffer cells (figure 1C; appendix pp 7–8). To distinguish serial uptake of bacteria from intracellular replication, mice were co-infected with K1-RM1628 and K2-GMR151. Microscopy data showed that clusters of bacteria were always monochrome, indicating a monoclonal infection localised to macrophages, providing a strong indication for bacterial intracellular replication (figure 1D). In-vitro controls (appendix pp 9, 22) showed that growth kinetics were comparable for all strains in medium, mouse serum, and whole blood (appendix p 9), indicating that clearance from the blood in vivo was probably dependent on tissue macrophage localisation. These data also showed that the mouse serum was not bactericidal, in line with whole-cell ELISA data, which found no evidence of *K pneumoniae-*specific antibodies (titre <32; appendix pp 24–25).

**Figure 2:**
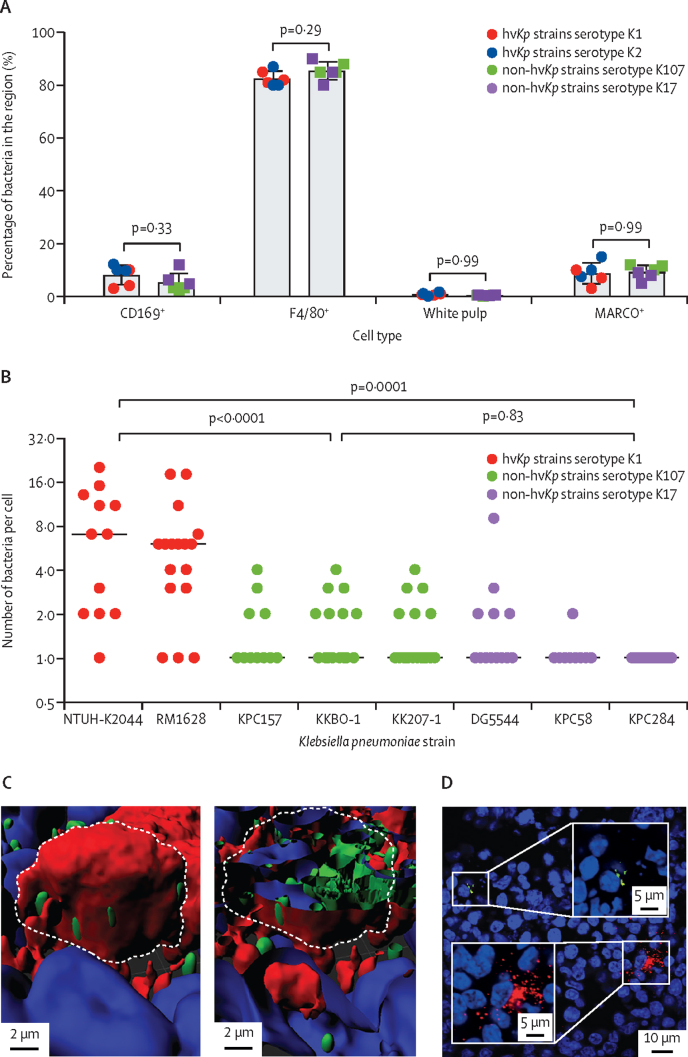
Tissue tropism and intracellular localisation of *Klebsiella pneumoniae* in mouse spleens following intravenous infection (A) Quantitative distribution of *K pneumoniae* between CD169^+^, F4/80^+^, MARCO^+^ macrophages, and the white pulp in the mouse spleen. Data are representative of two entire tissue sections of approximately 1 × 2 cm per mouse. (B) Size of bacterial clusters associated with host cells at 6 h after infection. Statistical significance was determined by one-way ANOVA. 30 random fields of view were analysed with 60 × magnification from the Olympus confocal microscope. Symbols represent individual bacterial clusters. (C) 3D reconstruction of a K1 focus of infection Z-stack acquired by confocal microscopy showing the top of the cell layer, and a single cut into the cell. DAPI stain indicated in blue, CD169 in red, and K1 ST23 in green. (D) K1 and K2 co-infected tissue section stained with purified IgG raised against K1 (green) capsule, or K2 (red). Lower magnification co-localisation images for all serotypes, in addition to confocal Z-stacks are shown in the appendix (pp 10–11). DAPI=4′,6-diamidino-2-phenylindole. hv*Kp*=hypervirulent *K pneumoniae*.

In the spleen, most bacteria (mean 84·00% [SD 3·38]) regardless of hypervirulence were found associated to F4/80^+^ red pulp macrophages, with fewer associated to CD169^+^ (mean 6·76% [3·75]) and MARCO^+^ marginal zone macrophages (mean 9·00% [3·31]), and almost none (mean 0·64% [0·31]) to the white pulp (figure 2A). Representative co-localisation images are shown for K1, K2, K17, and K107 strains (appendix pp 10–11). No difference in macrophage tropism was observed between hv*Kp* and non-hv*Kp* strains. Quantification of the number of bacteria associated with single host cells showed that the hv*Kp* K1 strain macrophage infection burden was significantly larger (p<0·0001; one-way ANOVA with Tukey's multiple comparisons; appendix pp 4–5) than non-hv*Kp* strains (figure 2B). As in the liver, the foci in splenic macrophages consisted of a median of seven (IQR 9·5) and eight (3·0) bacteria for hv*Kp* isolates NTUH-K2044 and RM1628, and of one (0·3) bacterium for all non-hv*Kp* isolates. For the spleen, K2 strains were excluded from the analysis because of high bacterial counts in the blood, which would confound microscopy analysis of the splenic red pulp which is a major reservoir for blood (appendix pp 10–11). Confocal microscopy Z-stack and line-scan analysis confirmed that in the spleen, foci of all strains were within macrophages (representative images in figure 2C; appendix pp 11–12). As for the liver, analysis of K1 and K2 co-infected mice revealed only monochrome bacterial clusters implying intramacrophage replication (figure 2D).

**Figure 3:**
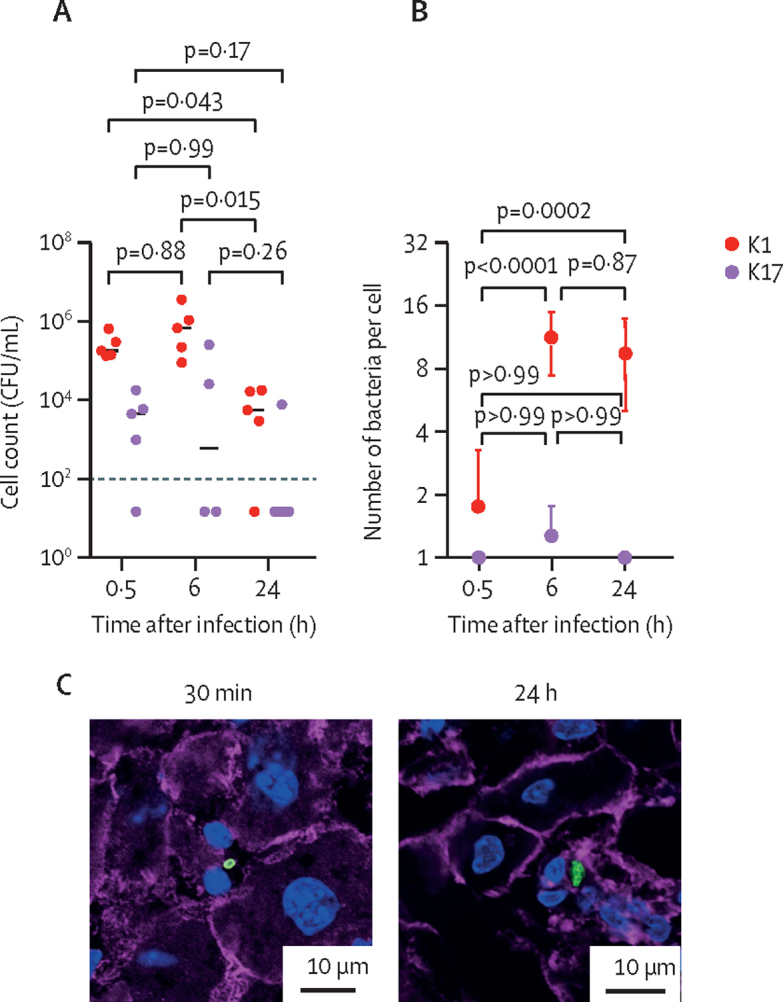
Time course analysis of K1 hypervirulent *Klebsiella pneumoniae* and K17 non-hypervirulent *K pneumoniae* growth and tissue distribution. (A) Bacterial counts are shown at 30 min, 6 h, and 24 h post-infection. The limit of detection is shown as a dotted black line. The median values are indicated by horizontal bars. Spleen counts are shown in the appendix (pp 12–13). (B) Size of host cell-associated bacterial clusters, of K1 and K17 *K pneumoniae* throughout the infection in the liver as determined from confocal microscopy. Data are representative of three mice per time point, per organ. Error bars indicate the standard error of the mean. (C) Representative images of the liver after infection with K1 and K17 *K pneumoniae* are shown for the 30 min and 24 h timepoints after infection. Nuclei are stained with DAPI in blue, *K pneumoniae* are green, and actin is stained with phalloidin in purple. CFU=colony forming unit. DAPI=4′,6-diamidino-2-phenylindole.

In our timecourse analysis of tissue infection in mice, the hv*Kp* K1 strain was cleared from the blood gradually over the first 24 h, whereas the non-hv*Kp* K17 strain was completely cleared from the blood by 30 min post-infection (figure 3A). Overall, the macrophage driven clearance of the infecting hv*Kp* strain was of approximately 80% by 6 h and 99% by 24 h after infection (figure 3A). The hv*Kp* strain had higher organ counts compared with the non-hv*Kp* strain at all-time points (figure 3A). In the case of the hv*Kp* strain, the size of the cell-associated bacterial foci in the liver increased markedly up to 6 h post-infection (mean 11·1 bacteria per Kupffer cell [SD 3·7]), but then plateaued in both the liver (figure 3B; representative images shown in figure 3C) and spleen (appendix pp 12–13). As expected, the non-hv*Kp* strain was found only as single bacteria associated with the phagocytes in both the liver (mean 1·3 bacteria per Kupffer cell [SD 0·5]) and spleen (mean 1·5 bacteria per cell [SD 0·5] appendix pp 12–13). At the 24 h timepoint, there was evidence of release of intracellular bacterial from infected cells into the surrounding area in the liver (appendix pp 14–15).

**Figure 4:**
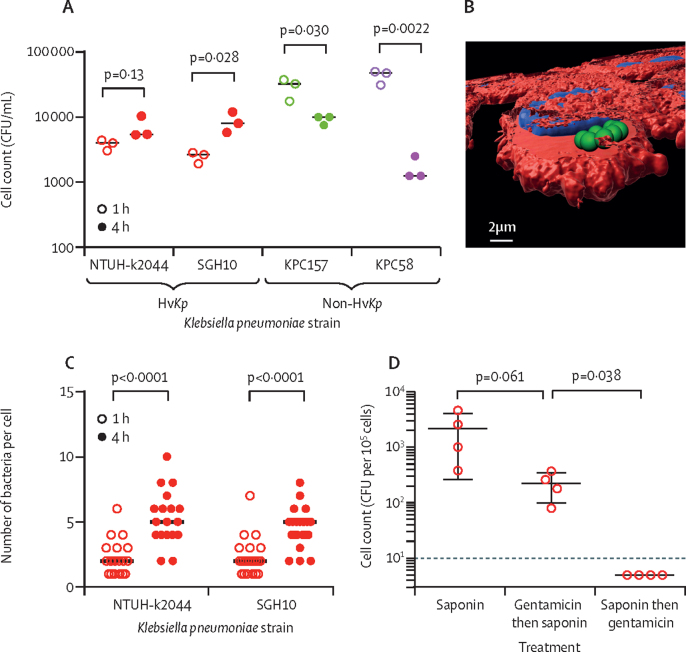
In-vitro characterisation of *Klebsiella pneumoniae* growth within macrophages (A) Gentamicin protection assay following infection of J774A.1 macrophages with K1 *K pneumoniae* strains NTUH-K2044 and SGH10 (red), K17 strain KPC157 (green) and K107 strain KPC58 (purple) at a multiplicity of infection of 10. (B) 3D reconstruction of confocal microscopy Z-stack of 4 h infected J774A.1 macrophages with *K pneumoniae* SGH10. Phalloidin are shown in red, *K pneumoniae* in green, and nuclei in blue. A single slice into the cell is shown indicating the bacteria's intracellular localisation. (C) Quantification of the number of bacteria associated with single cells infected as in part D in at least 15 fields of a 60 × magnification confocal microscope image. (D) Gentamicin protection assay done on liver cells taken ex vivo from mice intravenously infected with *K pneumoniae* SGH10. Error bars show the standard error of the mean. The dotted line indicates the limit of detection. CFU=colony forming unit. Hv*Kp*=hypervirulent *Klebsiella pneumoniae*.

From in-vitro infections of J774A.1 macrophages, we observed an increase in bacterial numbers within macrophages for K1 strain SGH10, but not for NTUH-K2044 from 1 h to 4 h after infection. However, we observed a decrease in bacterial counts for the K17 (KPC58) and K107 (KPC157) strains (figure 4A). Microscopically, at the 4 h time point, macrophages contained significantly more (p<0·0001; two-way ANOVA) *K pneumoniae* per cell (figure 4C), consistent with replication. 3D reconstructions of confocal Z-stacks confirmed that *K pneumoniae* were indeed intracellular in this in-vitro model (Figure 4B). To show bacteria were intracellular in the liver in vivo, we did a gentamicin protection assay of ex-vivo Kupffer cells from *K pneumoniae-*infected mice (figure 4D). This experiment showed that even after gentamicin treatment, live *K pneumoniae* could be cultured from Kupffer cells, confirming their intracellularity in vivo.

**Figure 5:**
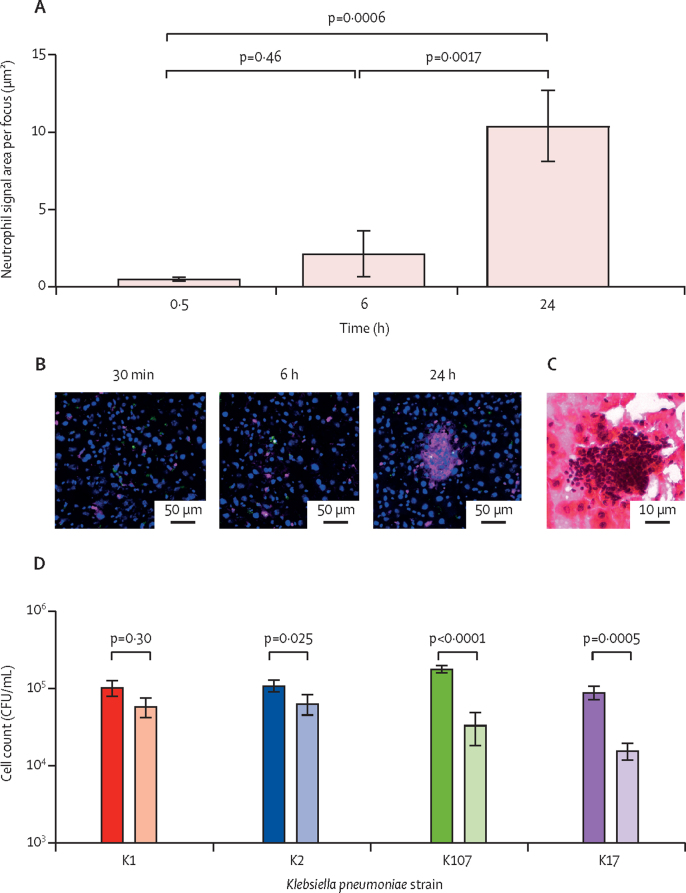
Neutrophil recruitment to bacterial foci in the liver leads to the formation of microabsesses (A) Quantification of neutrophil recruitment to infectious foci. Immunostained liver sections from 3 K1 *Klebsiella pneumoniae*-infected mice were quantified for neutrophil-associated fluorescence (relative fluorescence units) in a 25 μm radius around bacteria at 30 min, 6 h, and 24 h post-infection. Error bars indicate the standard deviation. (B) Representative image showing neutrophil recruitment to K1 foci at 30 min, 6 h, and 24 h after infection. Nuclei are stained with DAPI in blue, *K pneumoniae* are green, and neutrophils are pink. (C) Representative haematoxylin and eosin stain of mouse liver 48 h after infection with K1 *K pneumoniae*. (D) Neutrophil bactericidal assay of hypervirulent *K pneumoniae* (K1 and K2) and non-hypervirulent *K pneumoniae* (K107 and K17) showing bacterial counts before (filled bars) and after (open bars) a 2-h incubation with the neutrophils at a multiplicity of infection of 10. Error bars indicate the standard deviation. CFU=colony forming unit. DAPI=4′,6-diamidino-2-phenylindole.

In the liver, a gradual recruitment of neutrophils to hv*Kp* foci occurred over time (figure 5A, B). By 48 h after infection, neutrophils formed microabscesses around many of the hv*Kp* foci (Figure 5C), but not the non-hv*Kp* (appendix pp 14–15). At this stage, host cells associated with bacteria at the centre of the abscesses were negative for the macrophage marker F4/80, indicating absence of macrophages from the centres of abscesses (appendix pp 14–15). At 48 h, abscesses ranged in size in the tissue reaching a maximum area of 3000 μm^2^ with a median size of 252 μm^2^ (appendix pp 14–15). In the spleen, at 48 h after hv*Kp* infection, there was also evidence of neutrophil influx into the red pulp, but without the focal nature observed in the liver (appendix p 18). An ex-vivo neutrophil killing assay confirmed hv*Kp* to be more resistant to neutrophil-mediated killing than non-hv*Kp* (figure 5D).

**Figure 6:**
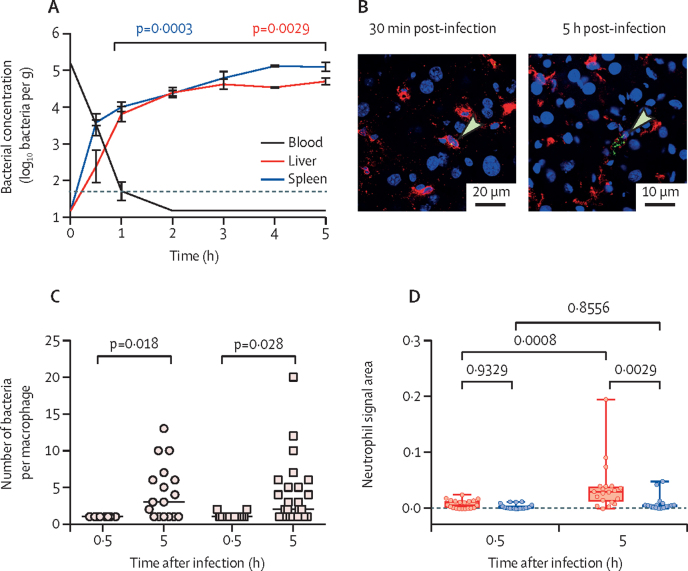
Ex-vivo infection of three independent perfused pig livers and spleens (A) Bacterial concentrations in pig blood, liver and spleen biopsies after infection of the perfusion circuit with 6·5 × 10^7^ CFUs of *Klebsiella pneumoniae* GMR151 K2-ST25. Data are representative of three independent perfusion experiments with individual data in the appendix (pp 18–19). Dotted line is the limit of detection. Statistical significance was determined using a *t* test. (B) Representative images of foci associated with CD169^+^ cells in the pig liver at 30 min and 5 h after infection. Nuclei are stained with DAPI (blue), CD169 cells are shown in red, *K pneumoniae* are shown in green, and arrows indicate bacteria. (C) Size of infectious foci (number of bacteria per macrophage) at 30 min and 5 h after infection in the pig liver (circles) and spleen (squares). Data were counted from confocal images of at least 20 macrophages per group. (D) Boxplots showing neutrophil signal area within a 50 μm radius of infected (red) or random non-infected (blue) macrophages, at 30 min and 5 h post-infection of the pig liver. Data are representative of entire tissue sections (approximately 2 cm^2^) from three replicate organs and were imaged at a consistent resolution. CFU=colony forming unit. DAPI=4′,6-diamidino-2-phenylindole.

We infected a novel ex-vivo perfused pig liver and spleen system with hv*Kp* and analysed serial biopsies from the same organ by quantitative culture and microscopy. After the first hour of perfusion the blood flow was steady (portal vein 0·11–0·27 L/min, hepatic artery 0·08–0·21 L/min, and splenic artery 0·06–0·13 L/min; appendix p 17). Following infection, hv*Kp* K2 ST25, bacteria were rapidly cleared from the circulating blood (figure 6A), but 30 min after infection, the liver contained a mean of 2·3 (SD 1·2) log_10_ bacteria per g of tissue and the spleen contained 3·5 (SD 0·2) log_10_ bacteria per g of tissue. Over time, the number of bacteria gradually increased in liver and spleen, to a maximum of 4·6 (SD 0·2) and 5·0 (SD 0·3) log_10_ bacteria per g, giving an approximate doubling time of 100 min. Bactericidal activity of serum, which had a median anti-GMR151 IgG titres of 512, contributed to the clearance of bacteria from the bloodstream (appendix pp 18–19).

In both the liver and the spleen at 30 min after infection, hv*Kp* were present as single cells or as pairs (figure 6B) associated with macrophages, whereas at 5 h post-infection, larger clusters of bacteria were observed in both organs (figure 6C). Confocal microscopy and 3D reconstruction confirmed that clusters of hv*Kp* were present intracellularly, in both the liver and the spleen (appendix pp 18–19).

To evaluate events preceding abscess formation in the pig organs, neutrophil recruitment to the sites of infection was investigated. Between 30 min and 5 h post-infection, the numbers of neutrophils significantly increased (p=0·0008; two-way ANOVA with Tukey's multiple comparisons; appendix pp 4–5) around infectious foci in the liver (figure 6D). By contrast, the number of neutrophils in non-infected areas remained unchanged. In the spleen, no significant neutrophil recruitment was observed in infected or uninfected tissue (appendix pp 20–21). Microscopy showed there was an overall increase in the number of neutrophils in the liver over time. In early biopsies, neutrophils were scattered throughout the liver tissue, whereas at 5 h after infection neutrophils clearly clustered around infectious foci in the liver. In the spleen, an increase of neutrophils was detected over time, but they remained localised to the red pulp surrounding the follicles (appendix pp 20–21).

## Discussion

Invasive *Klebsiella* infections associated with pyogenic liver abscesses, caused by serotype K1 and K2 hv*Kp* strains, are of increasing clinical concern^1^, ^3^, ^4^, ^16^ due to the recent descriptions of multidrug resistant hv*Kp* strains.^17^ We aimed to address the lack of understanding of the pathophysiology of the early stages of *Klebsiella* abscess formation in vivo. We show that diverse lineages of *K pneumoniae* can be distinguished by their ability to resist tissue macrophage-mediated killing, form a large tissue-restricted biomass, and subsequently resist neutrophil-mediated killing leading to abscess formation.

In this work, we showed that hv*Kp* strains differ from non-hv*Kp* by their ability to resist tissue macrophage-mediated killing in the mouse liver and spleen. Further, we provide evidence in our pig organ perfusion model, which enables serial sampling, that hv*Kp* might replicate within macrophages. This observation is contrary to the classical definition of *K pneumoniae* as an obligate extracellular pathogen, but it is in line with experiments with mouse alveolar macrophages and cell lines.^9^, ^10^ Although Kupffer cells have been shown to kill hv*Kp* in vitro,^18^ our data suggests that in vivo, Kupffer cells can sequester hv*Kp* but, depending on the conditions, either kill, as in the case of most phagocytosis events, or provide a niche for bacterial persistence. This phenotype of within-macrophage replication is consistently displayed by all hypervirulent *K pneumoniae* isolates, whereas other observed phenotypes such as bacteraemia levels or intracellular survival were found to be capsule type dependent or displayed by all isolates. These data are in accordance with observations that in alveolar macrophages the capsule was dispensable for survival of K2 hv*Kp*^9^ and that *K pneumoniae* might be replication competent within macrophages.^6^, ^19^ Cano and colleagues^9^ showed that the ability to resist macrophage mediated killing was probably due to the active inhibition of phagosome maturation, which resulted in no fusion of *K pneumoniae*-containing vacuoles with lysosomes. Further work will be required to understand whether this process occurs in splenic and hepatic tissue macrophage populations.

From the mouse infection model there was clear evidence for abscess formation in the liver, but not the spleen, exclusively after infection with hv*Kp*. This perfectly matches the clinical scenario in which infections with K1 and K2 hv*Kp* are characterised by abscesses in the liver and occasionally in other tissues including the spleen.^1^, ^16^, ^20^, ^21^, ^22^ *K pneumoniae* hepatic abscesses are characterised by an influx of neutrophils that are unable to control the local infection, leading to tissue damage,^23^ but events preceding this outcome have remained poorly characterised. Our data show that neutrophil recruitment is preceded by a period of resistance to macrophage-mediated clearance, which is in some cases associated with intracellular bacterial replication. At later stages, we observed bacteria being released from infected liver macrophages, an event associated with massive localised neutrophil influx. At this point, extracellular bacteria can be observed to be distributed across the maturing abscess, analogous to what occurs after staphylococcal infection.^24^ Surewaard and colleagues^25^ showed that *Staphylococcus aureus* replicating within Kupffer cells was undetectable by circulating neutrophils. Our data suggest a similar process, and we consequently propose a model whereby hv*Kp* can resist killing by Kupffer cells, initially evading detection by neutrophils, facilitating large macrophage infection burdens potentially exacerbated by intracellular replication. Later, these clusters lyse Kupffer cells, triggering inflammation and recruitment of neutrophils, which are unable to clear the extracellular biomass. The relative resistance of planktonic hv*Kp* to neutrophil killing^26^ is most likely to be exacerbated in the tissue, rendering bacterial aggregates fully resistant to neutrophil killing. Investigation of mechanisms underpinning release of bacteria from Kupffer cells (eg, apoptosis and passive lysis), in addition to the neutropenic mechanisms for tissue damage (eg, granule release) require investigation.

Infections of pig organs ex vivo provided compelling evidence for intracellular *K pneumoniae* replication in tissue macrophages in both liver and spleen, even in the presence of anti-*K pneumoniae* antibodies. Humans and mice vary in their immunology and macrophage biology^12^, ^27^, ^28^ but pig anatomy and immunology far more closely resemble the situation in humans, with comparable splenic and hepatic tissue macrophage architecture and blood neutrophil counts.^27^ The suitability of the pig as a model is underlined by the recent reports of epidemics of hv*Kp* K2 ST25 sepsis in pigs in the UK and Australia.^14^ Given that the mouse data are clearly replicated in this translational model, we propose that hepatic abscess formation in humans might also be preceded by a phase of within-macrophage replication of hv*Kp*, which results in neutrophil resistant microbial aggregates as the originating event of abscesses.

The main limitation of our study is the use of mouse infection models. The anatomical differences between mouse and human liver and spleen are only in part mitigated by translation to the pig ex-vivo perfusion model. We now have approval to study early events of infection in ex-vivo organ perfusion models of human spleen (ClinicalTrials.gov NCT04620824) and human liver segments (UK Health Research Authority Research Ethics Committee approval REC 21/PR/0287). These models should be ideally placed to translate the findings from this wovrk. The potential limitation of using an intravenous mouse infection model, by contrast to an oral infection model,^29^ might not be the route of administration—because translocation of bacteria from the gut to the liver also occurs through the portal vein—but because our model is limited to 48 h of infection because mice that developed abscesses tended to get septic, which limited the observation period.

Our study highlights the importance of hv*Kp* macrophage manipulation and the consequent pathophysiology leading to abscess formation, with potential implications for optimising antibiotic treatment regimens.^30^

## Data sharing

All original data and image files are available through the University of Leicester data repository Figshare (https://leicester.figshare.com/) at https://doi.org/10.25392/leicester.data.14443772.

## Declaration of interests

MP is a full-time employee of GlaxoSmithKline. GMR reports grants, personal fees, and non-financial support from Accelerate Diagnostics, Beckman Coulter, and Menarini; grants from Seegene, Arrow, Symcel, DID, Hain Lifescience, Meridian, SetLance, Qvella, Qlinea, Biomedical Service, and Quidel; personal fees from Becton Dickinson, Pfizer, Roche, Thermo Fisher, QPex, and Qiagen; grants and personal fees from bioMeriéux, Cepheid, MSD, Nordic Pharma, Shionogi, Zambon, and Angelini; personal fees from Venatorx; and income from selling strains to Venatorx, outside the submitted work. MRO reports a research contract with the University of Oxford, a studentship from the Medical Research Council (MRC), and grants from Royal Society Wolfson and the Biotechnology and Biological Sciences Research Council (BBSRC), during the conduct of the study; grants and a studentship from GlaxoSmithKline, outside the submitted work. All other authors declare no competing interests.

## References

[1] Siu LK, Yeh KM, Lin JC, Fung CP, Chang FY (2012). *Klebsiella pneumoniae* liver abscess: a new invasive syndrome. Lancet Infect Dis.

[2] Liu YC, Cheng DL, Lin CL (1986). *Klebsiella pneumoniae* liver abscess associated with septic endophthalmitis. Arch Intern Med.

[3] Fazili T, Sharngoe C, Endy T, Kiska D, Javaid W, Polhemus M (2016). *Klebsiella pneumoniae* liver abscess: an emerging disease. Am J Med Sci.

[4] Shon AS, Bajwa RPS, Russo TA (2013). Hypervirulent (hypermucoviscous) *Klebsiella pneumoniae*: a new and dangerous breed. Virulence.

[5] Nordmann P, Cuzon G, Naas T (2009). The real threat of *Klebsiella pneumoniae* carbapenemase-producing bacteria. Lancet Infect Dis.

[6] Bengoechea JA, Sa Pessoa J (2019). *Klebsiella pneumoniae* infection biology: living to counteract host defences. FEMS Microbiol Rev.

[7] Weiss G, Schaible UE (2015). Macrophage defense mechanisms against intracellular bacteria. Immunol Rev.

[8] Gordon S, Plüddemann A, Martinez Estrada F (2014). Macrophage heterogeneity in tissues: phenotypic diversity and functions. Immunol Rev.

[9] Cano V, March C, Insua JL (2015). *Klebsiella pneumoniae* survives within macrophages by avoiding delivery to lysosomes. Cell Microbiol.

[10] Fodah RA, Scott JB, Tam HH (2014). Correlation of *Klebsiella pneumoniae* comparative genetic analyses with virulence profiles in a murine respiratory disease model. PLoS One.

[11] Ercoli G, Fernandes VE, Chung WY (2018). Intracellular replication of *Streptococcus pneumoniae* inside splenic macrophages serves as a reservoir for septicaemia. Nat Microbiol.

[12] Chung WY, Wanford JJ, Kumar R (2019). An ex vivo porcine spleen perfusion as a model of bacterial sepsis. Altern Anim Exp.

[13] Chung WY, Gravante G, Al-Leswas D (2013). The development of a multiorgan ex vivo perfused model: results with the porcine liver-kidney circuit over 24 hours. Artif Organs.

[14] Bidewell CA, Williamson SM, Rogers J (2018). Emergence of *Klebsiella pneumoniae* subspecies pneumoniae as a cause of septicaemia in pigs in England. PLoS One.

[15] Lenth RV (2004). Power analysis for experimental research: a practical guide for the biological, medical and social sciences and statistical power analysis: a simple and general model for traditional and modern hypothesis tests. J Am Stat Assoc.

[16] Kong H, Yu F, Zhang W, Li X (2017). Clinical and microbiological characteristics of pyogenic liver abscess in a tertiary hospital in East China. Medicine (Baltimore).

[17] Gu D, Dong N, Zheng Z (2018). A fatal outbreak of ST11 carbapenem-resistant hypervirulent *Klebsiella pneumoniae* in a Chinese hospital: a molecular epidemiological study. Lancet Infect Dis.

[18] Hoh CH, Tan YH, Gan Y-H (2019). Protective role of Kupffer cells and macrophages in *Klebsiella pneumoniae* induced liver abscess disease. Infect Immun.

[19] Fodah RA, Scott JB, Tam HH (2014). Correlation of *Klebsiella pneumoniae* comparative genetic analyses with virulence profiles in a murine respiratory disease model. PLoS One.

[20] Arena F, Spanu T, Henrici De Angelis L (2016). First case of bacteremic liver abscess caused by an ST260-related (ST1861), hypervirulent *Klebsiella pneumoniae*. J Infect.

[21] Chang KC, Chuah SK, Changchien CS (2006). Clinical characteristics and prognostic factors of splenic abscess: a review of 67 cases in a single medical center of Taiwan. World J Gastroenterol.

[22] Lee CH, Hu TH, Liu JW (2005). Splenic abscess caused by *Klebsiella pneumoniae* and non-*Klebsiella pneumoniae* in Taiwan: emphasizing risk factors for acquisition of *Klebsiella pneumoniae* splenic abscess. Scand J Infect Dis.

[23] Fung CP, Chang FY, Lin JC (2011). Immune response and pathophysiological features of *Klebsiella pneumoniae* liver abscesses in an animal model. Lab Invest.

[24] Thammavongsa V, Missiakas DM, Schneewind O (2013). *Staphylococcus aureus* degrades neutrophil extracellular traps to promote immune cell death. Science.

[25] Surewaard BGJ, Deniset JF, Zemp FJ (2016). Identification and treatment of the *Staphylococcus aureus* reservoir in vivo. J Exp Med.

[26] Wang L, Shen D, Wu H, Ma Y (2017). Resistance of hypervirulent *Klebsiella pneumoniae* to both intracellular and extracellular killing of neutrophils. PLoS One.

[27] Steiniger BS (2015). Human spleen microanatomy: why mice do not suffice. Immunology.

[28] Dumigan A, Fitzgerald M, Santos JSG (2019). A porcine ex vivo lung perfusion model to investigate bacterial pathogenesis. MBio.

[29] Tu YC, Lu MC, Chiang MK (2009). Genetic requirements for *Klebsiella pneumoniae*-induced liver abscess in an oral infection model. Infect Immun.

[30] Dong N, Lin D, Zhang R, Chan EWC, Chen S (2018). Carriage of *bla*_KPC-2_ by a virulence plasmid in hypervirulent *Klebsiella pneumoniae*. J Antimicrob Chemother.

