# Supplementary material for: Interaction of Klebsiella pneumoniae with tissue macrophages in a mouse infection model and ex-vivo pig organ perfusions: an exploratory investigation
Source: Lancet Microbe. 2021 Dec;2(12):e695–703. doi: 10.1016/S2666-5247(21)00195-6 (PMC8641047; doi:10.1016/S2666-5247(21)00195-6)
Supplement: Supplementary appendix [file mmc1.pdf]

THE LANCET
Microbe

Supplementary appendix

This appendix formed part of the original submission and has been peer reviewed. We post it as supplied by the authors.

Supplement to: Wanford JJ, Hames RG, Carreno D, et al. Interaction of *Klebsiella pneumoniae* with tissue macrophages in a mouse infection model and ex-vivo pig organ perfusions: an exploratory investigation. *Lancet Microbe* 2021; published online Oct 18. https://doi.org/10.1016/S2666-5247(21)00195-6.

Supplementary materials

Interaction of Klebsiella pneumoniae with tissue macrophages in a mouse infection model and ex-vivo pig organ perfusions: an exploratory investigation

Joseph J. Wanford, Ryan G. Hames, David Carreno, Zydrune Jasiunaite, Wen Y. Chung, Fabio Arena, Vincenzo Di Pilato, Kornelis Straatman, Kevin West, Robeena Farzand, Mariagrazia Pizza, Luisa Martinez-Pomares, Peter W. Andrew, E. Richard Moxon, Ashley R. Dennison, Gian Maria Rossolini, Marco R. Oggioni.

INDEX

		Page
Table S1	Bacterial strains and metadata	2
Table S2	Antibodies and reagents used for immunohistochemistry	3
Table S3	Pipeline for statistical analysis in this manuscript	4
Figure S1	Fiji analysis pipeline for analysing distribution of bacteria in tissue	6
Figure S2	Low magnification image of multispectral images of liver section, and confocal Z-stacks used for 3D reconstructions	7
Figure S3	Growth kinetics of *Kp* strains following inoculation of rich medium BHI	9
Figure S4	Low magnification image of multispectral images of spleen section, and confocal Z-stacks used for 3D reconstructions	10
Figure S5	Foci size analysis of time course infections in the murine spleen	12
Figure S6	Evidence of Kupffer cell lysis following infection	14
Figure S7	Histological features of *Kp* infected mouse spleens	16
Figure S8	A normothermic model of liver-spleen co-perfusion for studies of *Kp*-host interactions	17
Figure S9	Independent replicates of *ex vivo* perfusion experiments	18
Figure S10	Microarchitecture of the porcine liver and spleen visible through multispectral imaging	20
Suppl. Methods		22
References		26

Table S1. Bacterial strains and metadata

Strain name	Serotype	ST	Source	hv*Kp* phenotype	Resistance phenotype	Carbapenemase	Virulence genes	Genome accession	Source	Ref
NTUH-K2044	K1: O1v2	23	Human, blood, Taiwan	+	-	-	*ybt, iuc, iro, rmpA, rmpA2*	AP006725.1	Pei-Fang Hsieh and Jin-Town Wang, Taipei, Taiwan	[1]
RM1628	K1: O1v2	1861	Human, blood, Italy	+	-	-	*ybt, clb, iuc, iro, rmpA, rmpA2*	JAALJC000000000	Teresa Spanu, Roma, Italy,	[2]
SGH10	K1: O1v2	23	Human liver abscess, Singapore	+	-	-	*ybt, clb, iuc, iro, rmpA, rmpA2*	NZ_CP025080.1	NCTC	[3]
GMR151	K2: O1v2	25	Human, blood, Italy	+	ESBL	-	*iuc, iro, rmpA, rmpA2*	JAALJD000000000	Rossana Cavallo, Torino, Italy	N/A
HMV-1	K2: O1v1	86	Human, blood, Italy	+	-	-	*ybt, iro, rmpA*	JAALCW000000000	Rossolini, Florence, Italy	N/A
HMV-2	K2: O1v2	65	Human, blood, Italy	+	-	-	*iuc, iro, rmpA, rmpA2*	JAALCV000000000	Rossolini, Florence, Italy	N/A
KPC157	KL107: O2v2	512	Human, rectal swab, Italy	-	CR	bla_{KPC-3}	-	JAALCT000000000	Rossolini, Florence, Italy	N/A
KKBO-1	KL107: O2v2	258	Human, blood, Italy	-	CR	bla_{KPC-3}	-	GCA_000495875.1	Rossolini, Florence, Italy	[4]
KK207-1	KL107: O2v2	258	Human, blood, Italy	-	CR	bla_{KPC-2}	-	GCA_001399815.1	Rossolini, Florence, Italy	[5]
HS11286	KL103: O2v2	11	Human, sputum, China	-	CR	bla_{KPC-2}	*ybt*	NC_016845.1	Hong-Yu Ou, Shanghai	[6]
DG5544	K17: O1v1	2502	Human, rectal swab, Italy	-	CR	bla_{KPC-3}	*ybt*	GCF_003227695.1	Rossolini, Florence, Italy	[7]
KPC58	K17: O1v1	101	Human, blood, Italy	-	CR	bla_{KPC-2}	*ybt*	JAALCT000000000	Rossolini, Florence, Italy	N/A
KPC284	K17: O1v1	101	Human, blood, Italy	-	CR	bla_{KPC-2}	*ybt*	JAALCS000000000	Rossolini, Florence, Italy	[8]

Abbreviations: ESBL, extended-spectrum β-lactamase; CR, carbapenem-resistant; *ybt*, yiersiniabactin; *iuc,* aerobactin; *iro*,salmochelin; *clb*, colibactin; *rmpA*, regulator of mucoid phenotype;

Table S2. Antibodies and reagents used for immunohistochemistry

Antibody/reagent (clone)	Target	Dilution	Conjugate	Catalogue	Supplier
Rat anti-mouse CD169 (3D6.112)	CD169+ MΦ	1:200	None	MCA884GA	Biorad, USA; Ca
Rat anti-mouse F4/80 (A3-1)	RP MΦ/ Kupffer cells	1:200	None	MCA497RT	Biorad, USA; Ca
Rat anti-mouse MARCO (ED31)	MZM MΦ	1:200	None	MCA1849T	Biorad, USA; Ca
Rabbit anti-K1 antisera	*Kp* serotype K1	1:500	None	N/A	SSI, Denmark, København
Rabbit anti-K2 antisera	*Kp* serotype K2	1:500	None	N/A	SSI, Denmark, København
Rabbit anti-pan *Klebsiella*	*Kp*	1:200	None	aBii0947	Abcam, UK; Cambridge
Rat anti-mouse Ly-6G (1A8)	Neutrophils	1:200	None	MCA711A647	Biorad, USA; Ca
Mouse anti-porcine CD169 (3BI1/11)	CD169+ MΦ	1:200	None	MCAII316GA	Biorad, USA; Ca
Mouse anti-porcine CD163 (2AI0/11)	CD163+ MΦ	1:200	None	MCAII311GA	Biorad, USA; Ca
Mouse anti-porcine granulocytes (2BII)	Granulocytes	1:200	None	MCAII600GA	Biorad, USA; Ca
Goat anti-rat IgG - 647	Rat primary antibody	1:500	Alexafluor 647	A-21247	Thermo, USA, Ma
Goat anti-rat IgG - 568	Rat primary antibody	1:500	Alexafluor 568	A-11077	Thermo, USA, Ma
Goat anti-rabbit IgG - 488	Rabbit primary antibody	1:500	Alexafluor 488	A-11008	Thermo, USA, Ma
Goat anti-mouse IgG - 568	Mouse primary antibody	1:500	Alexafluor 568	A-21124	Thermo, USA, Ma
Phalloidin	Actin	1:100	Alexafluor 647	AII2287	Thermo, USA, Ma
Texas red conjugation kit	N/A	N/A	Texas red	aBi95225	Abcam, UK; Cambridge
FITC conjugation kit	N/A	N/A	FITC	aBi02884	Abcam, UK; Cambridge
DAPI	Nuclei	N/A	DAPI-405	D9542	Sigma, USA; Mo

MΦ; macrophage, RP; red pulp, MZM; marginal zone macrophages, N/A; not applicable; SSI Statens Serum Institut

Table S3. Pipeline for statistical analysis in this manuscript

Figure	Test	Measurement	Comparison	P value
1a	2-way ANOVA with Tukey's multiple comparisons	Fluorescence co-localisation of bacteria with host cells	HV vs non-HV in F4/80+ cells	0.9665
			HV vs non-HV F4/80- cells	0.9665
1b	Ordinary 1-way ANOVA with Tukey's multiple comparisons	Size of cell-associated bacterial clusters in confocal microscopy images	K1 vs K2	0.7024
			K1 vs K107	<0.0001
			K1 vs K17	<0.0001
			K2 vs K107	0.0003
			K2 vs K17	0.0002
			K107 vs K17	0.9928
2a	2-way ANOVA with Tukey's multiple comparisons	Fluorescence co-localisation of bacteria with host cells	HV vs non-HV CD169+ cells	0.2267
			HV vs non-HV F4/80+ cells	0.2953
			HV vs non-HV White pulp	0.9983
			HV vs non-HV MARCO+ cells	0.9972
2b	Ordinary 1-way ANOVA with Tukey's multiple comparisons	Size of cell-associated bacterial clusters in confocal microscopy images	K1 vs K17	<0.0001
			K1 vs K107	<0.0001
			K17 vs K107	0.8309
3a	2-way ANOVA with Tukey's multiple comparisons	Bacterial CFU recovered from homogenised tissue after infection	K1 blood, 30m vs 6h	0.0009
			K1 blood, 6h vs 24h	0.0435
			K1 blood, 30m vs 24h	<0.0001
			K17 blood, 30m vs 6h	>0.9999
			K17 blood, 6h vs 24h	>0.9999
			K17 blood, 30m vs 24h	>0.9999
			K1 liver, 30m vs 6h	0.6795
			K1 liver, 6h vs 24h	0.8516
			K1 liver, 30m vs 24h	0.3582
			K7 liver, 30m vs 6h	0.5362
			K7 liver, 6h vs 24h	>0.9999
			K7 liver, 30m vs 24h	0.5292
3b	2-way ANOVA with Tukey's multiple comparisons	Size of cell-associated bacterial clusters in confocal microscopy images	K1, 30m vs 6h	<0.0001
			K1, 6h vs 24h	0.8573
			K1, 30m vs 24h	0.0002
			K17, 30m vs 6h	>0.9999
			K17, 6h vs 24h	>0.9999
			K17, 30m vs 24h	>0.9999
4a	Unpaired t test	Intracellular bacteria recovered from infection cells	NTUH-k2044, 1h to 4h PI	0.1307
			SGH10, 1h to 4h PI	0.0288
			KPC157, 1h to 4h PI	0.03
			KPC58, 1h to 4h PI	0.0022
4c	2-way ANOVA with Sidak's multiple comparisons	Size of cell-associated bacterial clusters in confocal microscopy images	NTUH-k2044, 1h to 4h PI	<0.0001
			SGH10, 1h to 4h PI	<0.0001
4d	Ordinary 1-way ANOVA with Dunnett's multiple comparisons	Bacteria recovered from *ex vivo* liver cells after treatment	Saponin vs gentamicin treatment followed by saponin	0.0606
			Saponin vs saponin followed by gentamicin treatment	0.0385
5a	Ordinary 1-way ANOVA	Neutrophil fluorescence signal area by confocal microscopy	0.5 vs 24h PI	0.0006
			6 vs 24h PI	0.0017
			0.5 vs 6h PI	0.4631
5d			K1	0.0303

	2-way ANOVA with Tukey’s multiple comparisons	Bacteria recovered after 30 min incubation with *ex vivo* murine neutrophils	K2	0.0258
			K107	<0.0001
			K17	0.0005
6a	Unpaired t test	Bacteria recovered from homogenised organ biopsy	Spleen, 0.5 to 5h PI	0.0003
			Liver, 0.5 to 5h PI	0.0029
6c	Unpaired t test	Size of cell-associated bacterial clusters in confocal microscopy images	Spleen, 0.5 to 5h PI	0.0282
			Liver, 0.5 to 5h PI	0.0188
6d	2-way ANOVA with Tukey’s multiple comparisons	Neutrophil signal area in confocal microscopy images	5h infected vs UI	0.0029
			0.5h infected vs UI	0.9329

PI; post infection

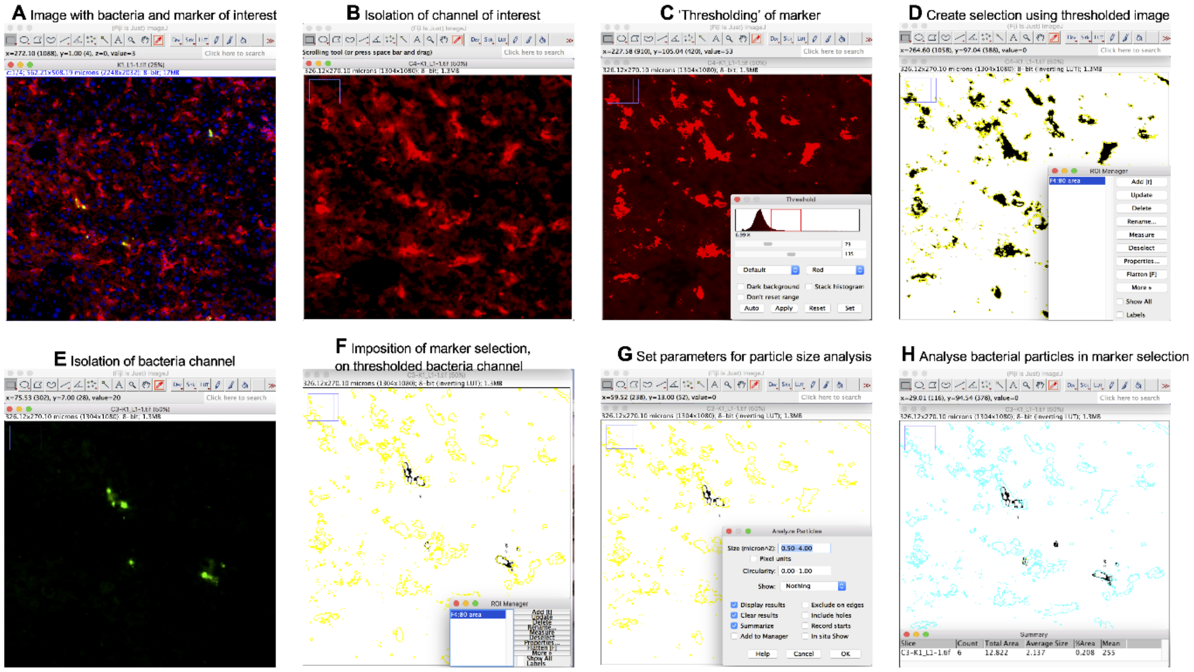

$$\% \text{ co-localization} = \frac{\mathit{Thresholded\ bacterial\ fluorecence\ area\ in\ cellular\ ROI}}{\mathit{Total\ thresholded\ bacterial\ fluorecence\ area}} \text{ x } 100$$

Figure S1. Fiji analysis pipeline for analysing distribution of bacteria in tissue. (A) First, a multi-colour channel image is required which includes a bacterial stain, and a stain which delineates a marker of interest. (B) Next the channel which identifies the cellular marker should be isolated using the split channels function, before (C) thresholding the image to highlight only the cell area above an arbitrary fluorescence threshold. (D) This thresholded images is then used to produce a region of interest (roi) selection. (E-F) This selection can then be applied to the bacterial fluorescence channel, before particle size analysis is performed (G-H) with suitable parameters for the bacterium of interest.

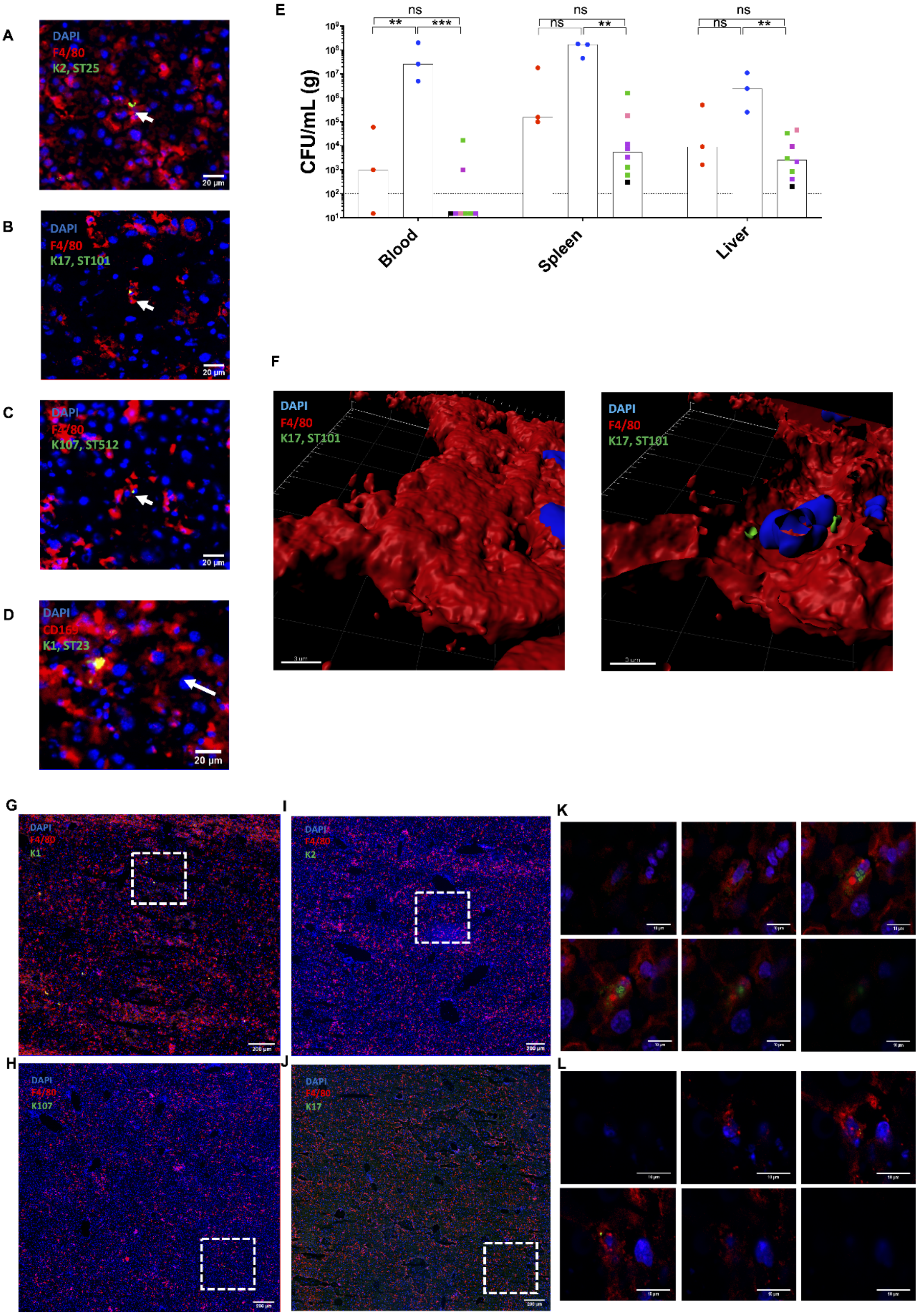
A
DAPI
F4/80
K2, ST25
20 µm
B
DAPI
F4/80
K17, ST101
20 µm
C
DAPI
F4/80
K107, ST512
20 µm
D
DAPI
CD169
K1, ST23
20 µm
E
ns
**
***
ns
ns
**
ns
ns
**
CFU/mL (g)
Blood
Spleen
Liver
F
DAPI
F4/80
K17, ST101
DAPI
F4/80
K17, ST101
G
DAPI
F4/80
K1
200 µm
I
DAPI
F4/80
K2
200 µm
K
H
DAPI
F4/80
K107
J
DAPI
F4/80
K17
L

Figure S2. Low magnification image of multispectral images of liver section, confocal Z-stacks used for 3D reconstructions, and bacterial titres in the tissue after intravenous infection. Samples are stained with DAPI, shown in blue, F4/80 in red, and antibodies targeted against *Kp* K2 (A), K17 (B), KL107 (C), and K1 (D) shown in green in each case. The scale bar is indicated in the bottom right of each image. (E) Bacterial counts in blood, spleen and liver homogenates after 6h intravenous infection with individual strains of *Kp.* Strains are coloured by serotype, red; K1, blue; K2, purple; K17, green; K107. Data are expressed as CFU/mL of blood, or CFU/G of homogenised tissue. Statistical significance was determined by 2-way ANOVA. (F) 3D reconstructions of confocal Z-stacks showing K17 *Kp* in the confines of an F4/80 membrane stain. Low magnification scanning microscopy images of K1 (G), K2 (H), K107 (I), and K17 (J) *Kp* corresponding to higher magnification images in Figure 1 and Figures S2A-C*.* The areas presented in the aforementioned figures are outlined by dotted white lines. Z-stack images of K1 (K) and K17 (L) infected F4/80+ (red) macrophages. Sections are shown at 2um optical plane intervals from the top of the tissue section (top left) to the bottom of the tissue section (bottom right). These Z stacks were used to produce the 3D reconstructions shown in Figure 1. The scale bars for these images are 10um in width.

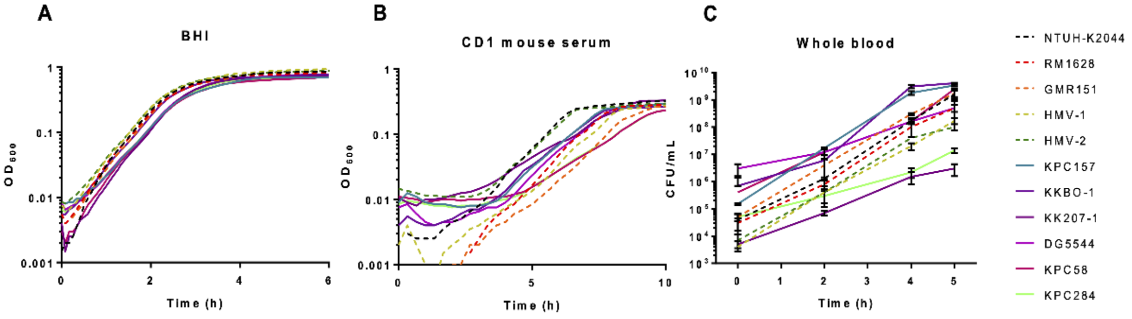

Figure S3. Growth kinetics of *Kp* strains following inoculation of rich medium BHI (A), *ex vivo* CD1 mouse sera (B), and *ex vivo* CD1 whole mouse blood (C) with ~10^5 CFU of each strain. hv*Kp* strains are shown by dotted lines, whereas non-hv*Kp* strains are shown by solid lines. Strain names corresponding to the colour of each strain are shown to the right of the graph.

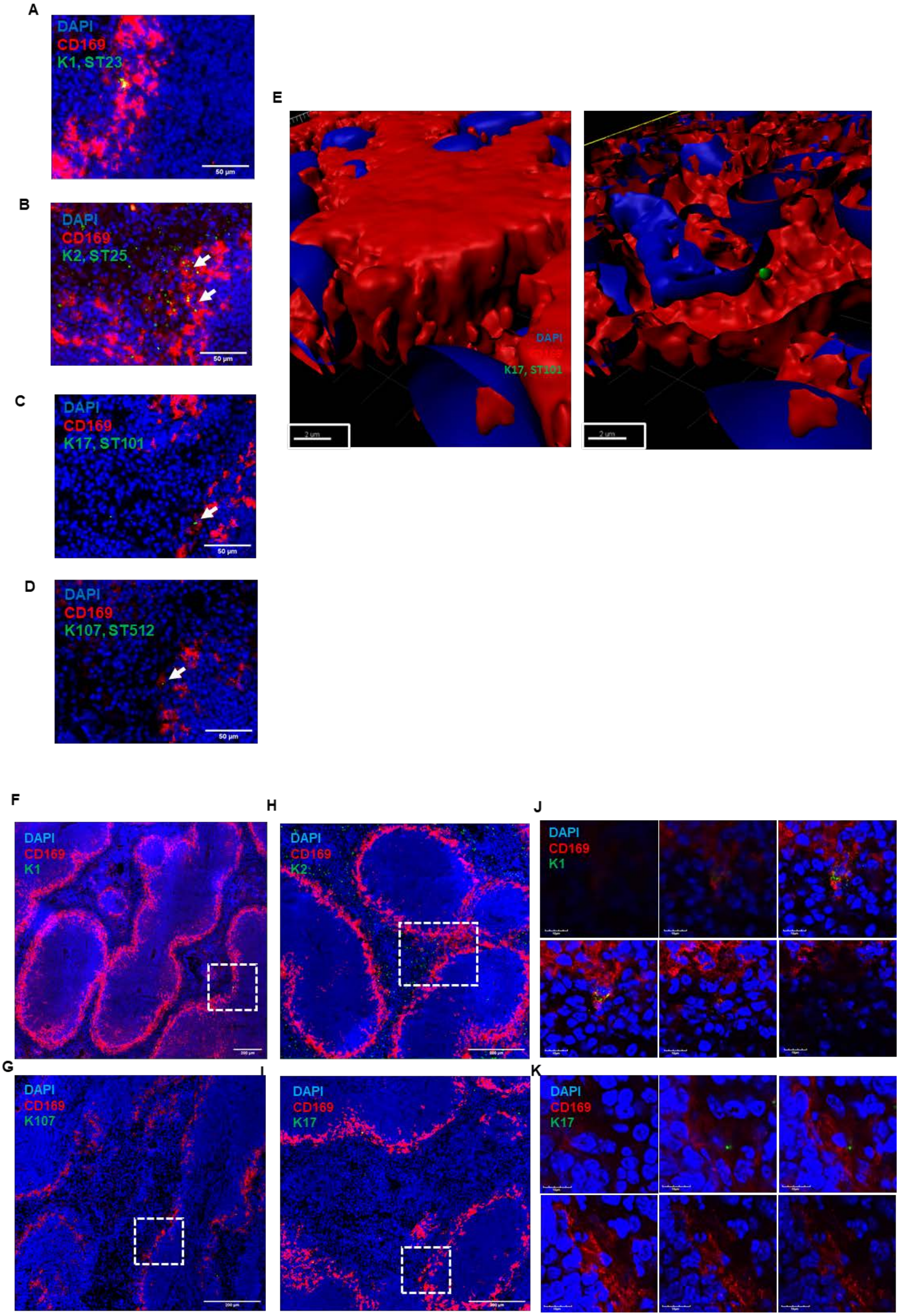

Figure S4. Low magnification image of multispectral images of spleen section, and confocal Z-stacks used for 3D reconstructions. Samples are stained with DAPI, shown in blue, CD169 in red, and antibodies targeted against *Kp* K1 (A), K2 (B), K17 (C), KL107 (D),

shown in green in each case. The scale bar is indicated in the bottom right of each image. (E) 3D reconstructions of confocal Z-stacks showing K17 *Kp* in the confines of an CD169 membrane stain. Low magnification scanning microscopy images of K1 (F), K2 (G), K107 (H), and K17 (I) *Kp* corresponding to higher magnification images in Figure 2 and Figures S4A-C. The areas presented in the aforementioned figures are outlined by dotted white lines. Z-stack images of K1 (J) and K17 (K) infected CD169+ (red) macrophages. Sections are shown at 2um optical plane intervals from the top of the tissue section (top left) to the bottom of the tissue section (bottom right). These Z stacks were used to produce the 3D reconstructions shown in Figure 1. The scale bars for these images are 10um in width.

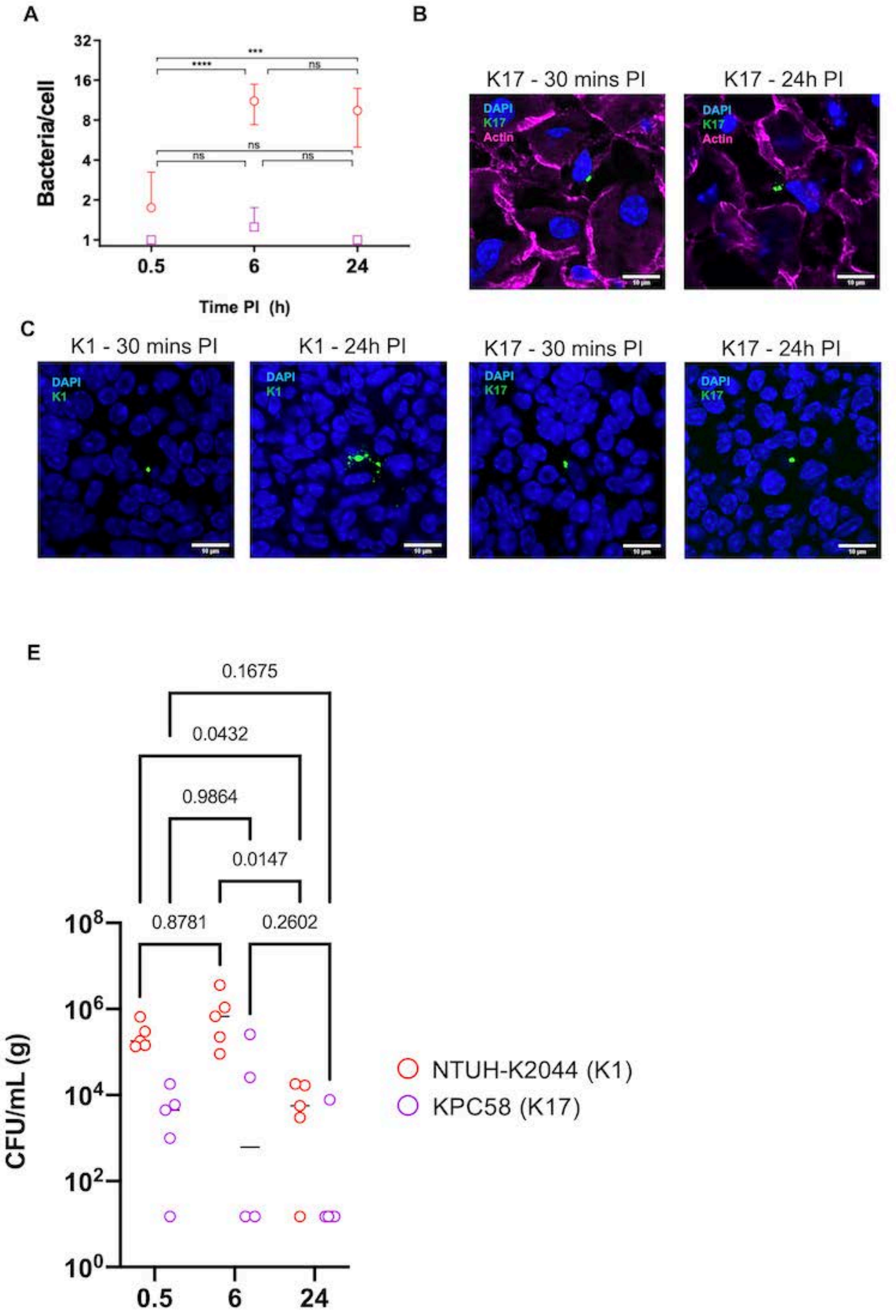

Figure S5. Foci size analysis of time course infections in the murine spleen. (A) Size of bacterial clusters associated with host cells in mouse spleens infected with either K1 (red) or K17 (purple) *Kp* at 0.5, 6, and 24h post infection. Error bars represent the standard error of the mean. Statistical comparison was performed by 1-way ANOVA. ****; p < 0.00005, ***; p < 0.0005, ns; not significant. (B) Representative confocal microscopy images of K17 infected livers at 0.5 and 24 hours post infection. *Kp* are shown in green, actin in magenta, and nuclei

in blue. (C) Representative confocal microscopy images of K1 and K17 infected spleens at 0.5 and 24 hours post infection. *Kp* are shown in green, and nuclei in blue. (D) Bacterial counts in the murine spleen after intravenous infection with 10^6 CFU of K1 (red) or K17 (magenta) *Kp.* Values are expressed as CFU/g of organ. Statistical analysis was performed by 2-way ANOVA. *; p > 0.05, ns; not significant.

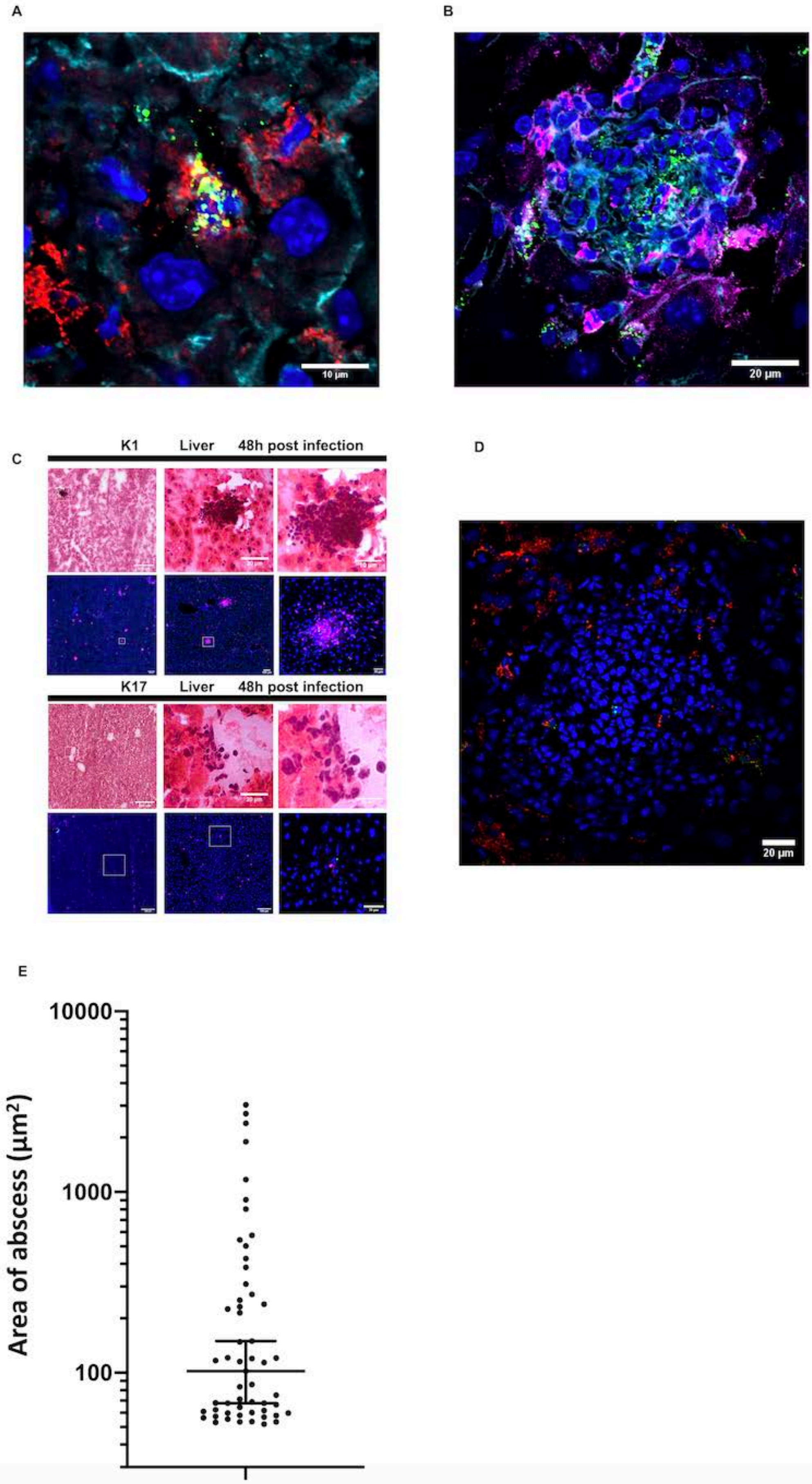
A
B
10 µm
20 µm
C
K1 Liver 48h post infection
K17 Liver 48h post infection
D
20 µm
E
10000
1000
100
Area of abscess (µm²)

Figure S6. Evidence of Kupffer cell lysis following infection. Mice were infected for 24h with 10^6 CFU of *Kp* strain NTUH-K2044, and their livers were analysed by confocal microscopy followed by staining with different antibodies. (A) Shows a Kupffer cell which is heavily infected with *Kp*, such that the bacteria extend across the cell into the neighbouring area. Nuclei; blue, *Kp*; green, CD169; red, Actin; cyan. (B) Shows a maturing abscess with *Kp* spread across multiple cells in many cases co-localising with neutrophils. Nuclei; blue, *Kp*; green, Ly-6G; magenta. (C) Representative H&E stains, and accompanying neutrophil IHC of mouse liver following 48h of infection with either with K17 *Kp.* 3 levels of increasing magnification are shown from left to right. (D) F4/80 immunostain of murine liver infected with K1 *Kp* at 48h post infection showing the absence of macrophages in the dense PMN cell centre. (E) Area in micron squared of neutrophil fluorescence signal in immunostained liver from mice infected for 48h with hv*Kp.* A cut-off value at 85 μm^2 excluding single neutrophils was introduced (dotted line); median and confidence values are indicated in the graph. Error bars show the standard deviation.

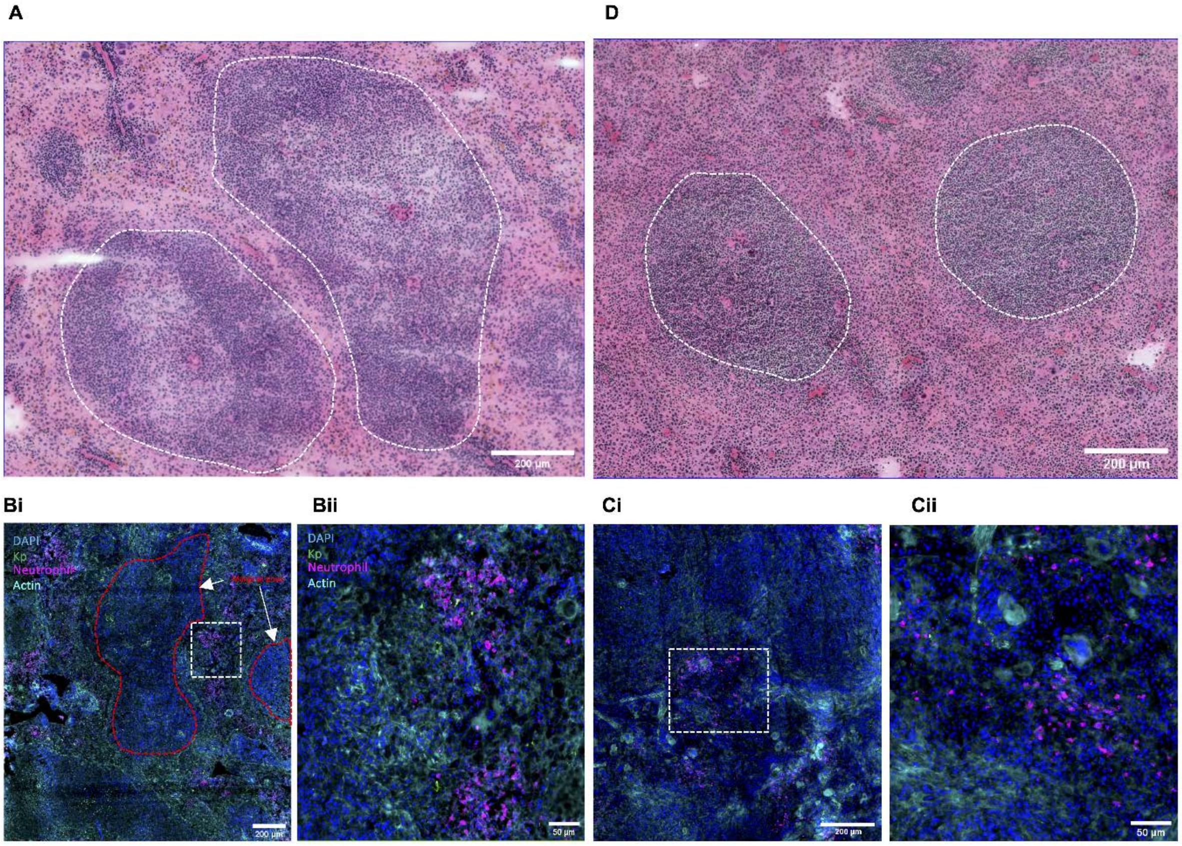

Figure S7. Histological features of *Kp* infected mouse spleens. H&E stain of a mouse spleen infected for 48h with hv*Kp* strain NTUH-K2044 (A) or with the non-hv*Kp* strain KPC58 (B). In both images the border between the splenic white pulp and red pulp are shown by a dotted white line. For both images, the scale bar is 200 µm in width. (Bi) Fluorescence image of the murine spleen infected for 48h with hv*Kp.* The border of the splenic white pulp is shown by a dotted red line. A dotted white line indicates an infected area, which is shown at higher magnification in Bii. (Ci) Fluorescence image of the murine spleen infected for 48h with non-hv*Kp.* A dotted white line indicates an infected area, which is shown at higher magnification in Cii. For Bi and Ci, the scale bar is 200 µm in width. For Bii and Cii, the scale bar is 50 µm in width. All samples are stained for nuclei (blue), bacteria (green), neutrophils (magenta) and actin (cyan).

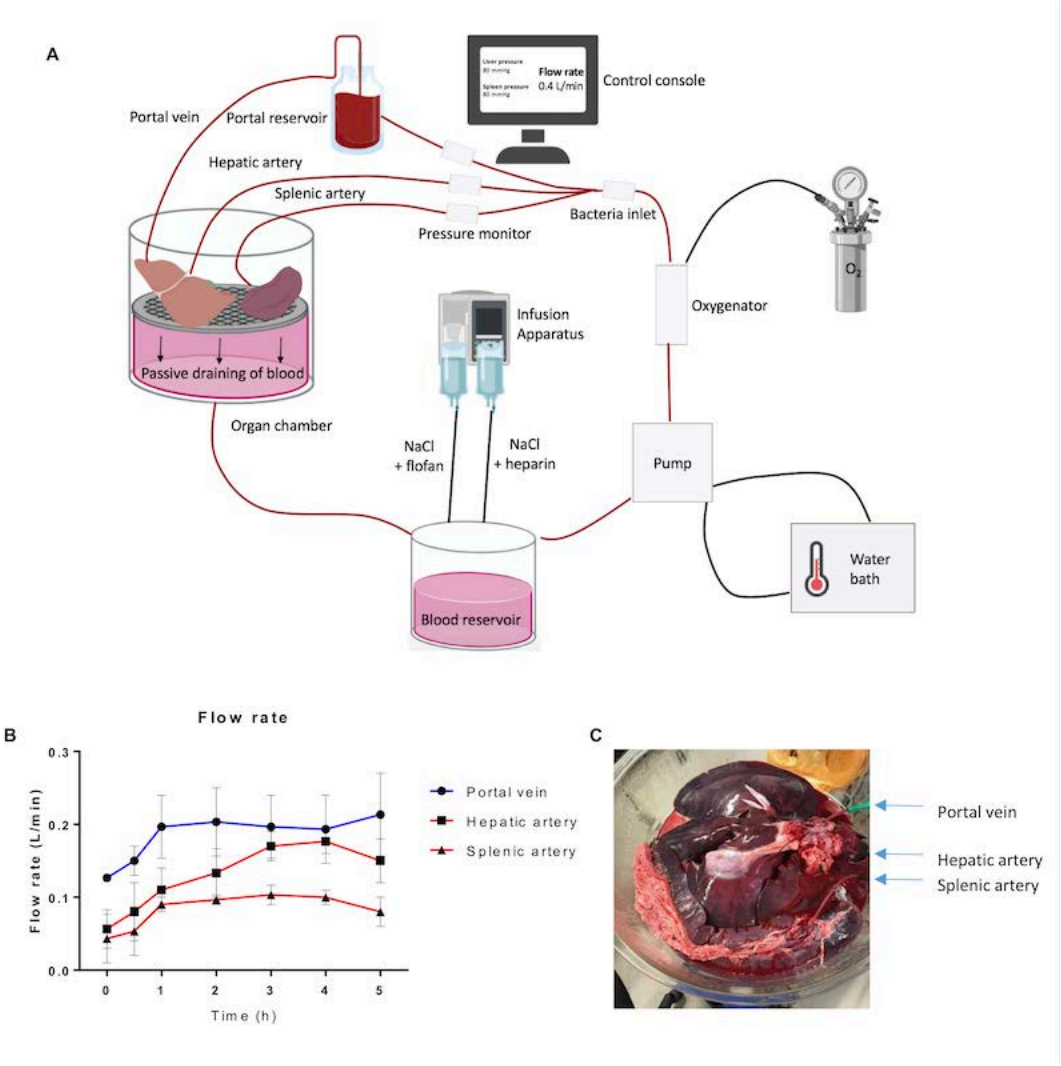

Figure S8. **A normothermic model of liver-spleen co-perfusion for studies of *Kp*-host interactions.** (A) The circuit consists of a chamber which holds both spleen (cannulated via the splenic artery) and liver (cannulated by the hepatic artery and portal vein). Blood drains from this chamber into a blood reservoir which is fed with Flofan (vasodilator) and Heparin (anticoagulant) solutions. From here, blood is pumped through a water bath which maintains a stead 37oc temperature, and then through an oxygenator. At this point, there is an inlet for bacterial inoculation, before the circuit splits into 3 vessels: the splenic artery, the hepatic artery, and the portal vein via an additional blood reservoir. Each of these vessels is monitored for pressure, which is relayed on the control console. (B) Flow rate is monitored for each vessel at regular time points. The portal vein is shown by black circles with a blue line, the hepatic artery is shown by black squares with a red line, and the splenic artery is shown by black triangles with a red line. (C) Shows a photograph of the organs *in situ*, with the cannulated portal vein, splenic artery, and hepatic artery indicated by blue arrows.

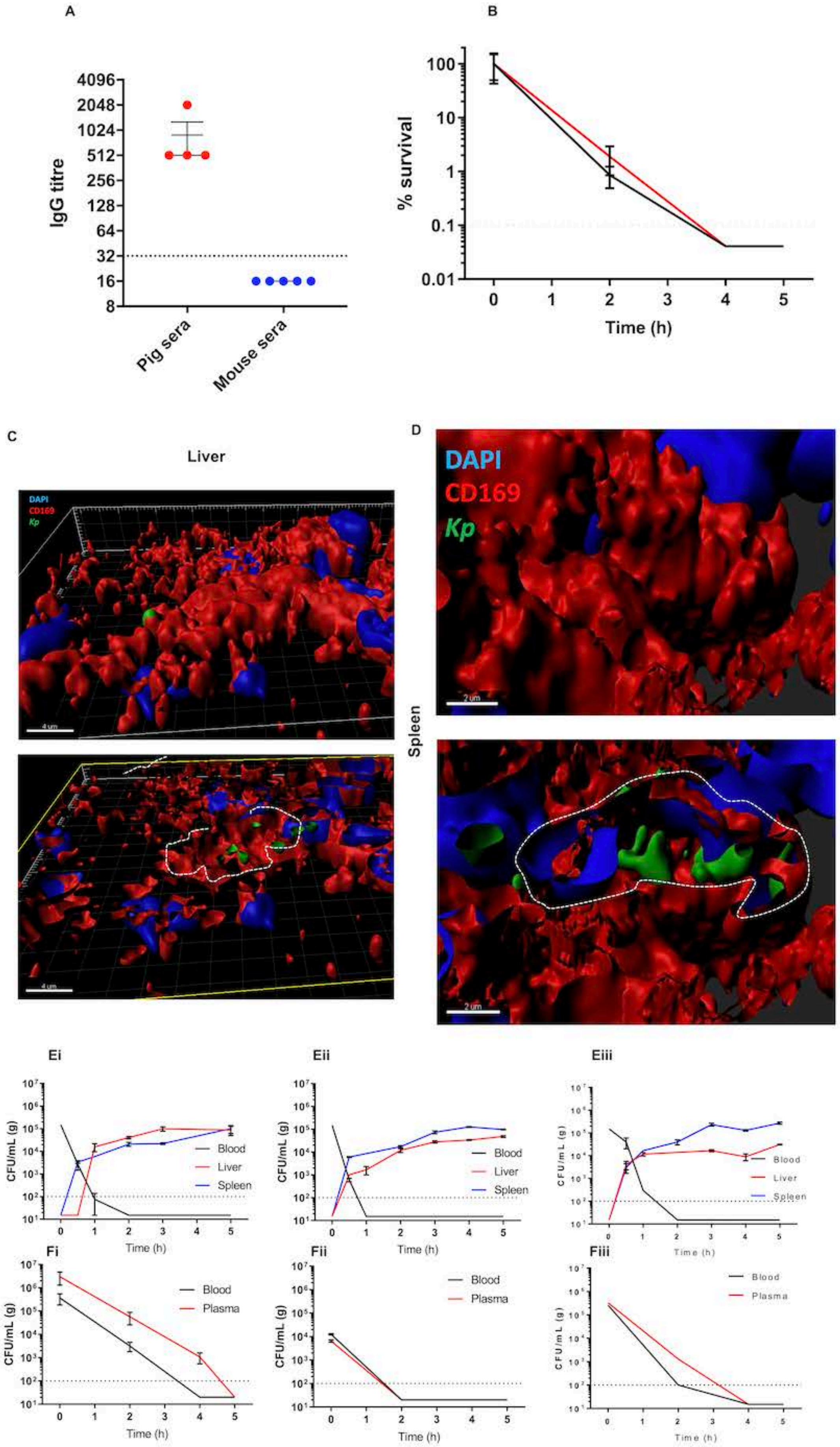
A
IgG titre
Pig sera
Mouse sera
B
% survival
Time (h)
C
Liver
DAPI
CD169
Kp
D
Spleen
DAPI
CD169
Kp
Ei
Eii
Eiii
CFU/mL (g)
Blood
Liver
Spleen
Time (h)
Fi
Fii
Fiii
Blood
Plasma

Figure S9. (A) IgG titres of murine and porcine sera used in this study against K2 *Kp* GMR151. Titres were defined as the reciprocal of the last dilution giving signal above the negative control. (B) Whole pig blood, and serum survival of bacteria following inoculation of ~5x10^5 CFU of *Kp* K2 GMR151. Blood counts are shown in black, and plasma counts are shown in red. (C) Representative 3D reconstruction of Z-stacks of *Kp* infected liver and splenic (D) macrophages in the porcine model. The cell border is indicated with a dotted white line. (Ei-iii) 3 independent replicates of CFU data from ex vivo perfusion experiments. Blood counts are shown in black, liver counts in red, and spleen counts in blue. The limit of detection is shown by a dotted black line. The error bars indicate the standard deviation of 3 technical replicates. (Fi-iii) 3 independent replicates of whole pig blood, and serum survival of bacteria following inoculation of ~5x10^5 CFU of *Kp* K2 GMR151. Blood counts are shown in black, and plasma counts are shown in red. (C) IgG titres of murine and porcine sera used in this study against K2 *Kp* GMR151. Titres were defined as the reciprocal of the last dilution giving signal above the negative control.

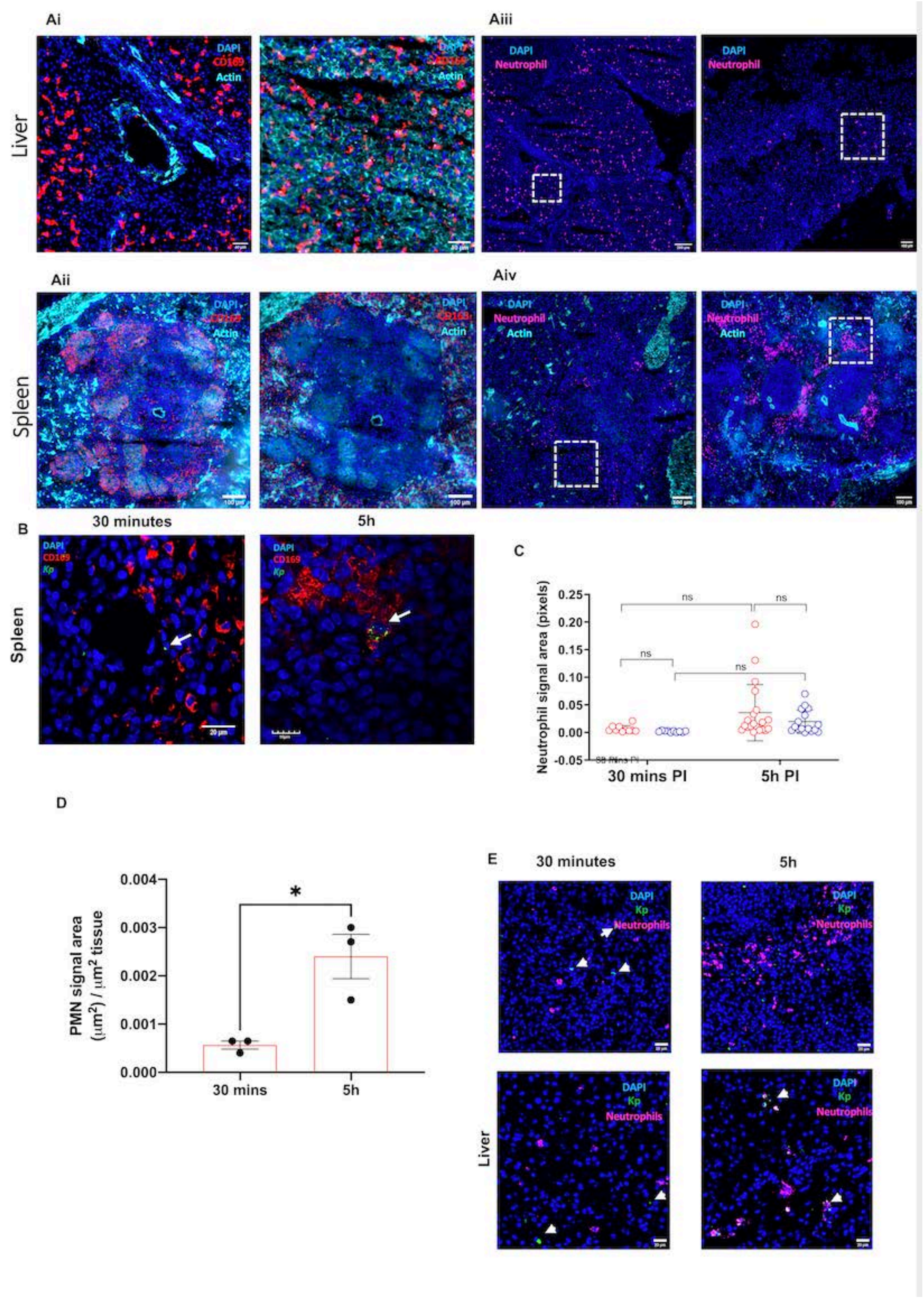

Figure S10. Microarchitecture of the porcine liver and spleen visible through multispectral imaging. (Ai) Images of a hepatic vessel at the intersection of 3 porcine liver lobuli, Kupffer cell distribution in the parenchyma of the porcine liver. Both sections are stained for nuclei (blue), CD169 (red), and actin (cyan). In both, scale bars are 40 um in width. (Aii) Low magnification image of a porcine splenic follicle, with the major artery in the centre of the image, stained for nuclie (blue), actin (cyan), and either CD169 (red) which identifies the peri-

arteriolar lymphoid sheathe macrophages, or CD163 (red) which delineated the splenic red pulp macrophages. Low magnification image of the porcine liver (Aiii) and spleen (Aiv) 30 minutes or 5h post infection with GMR151, stained for neutrophils (magenta), nuclei (blue), and *Kp* (green). In each image, the area from which the high magnification image in Figure 6 is indicated by a dotted white line. (B) representative images of bacteria clusters in *Kp* infected porcine spleens at 30 minutes and 5h post infection. CD169 are shown in red, *Kp* in green, and nuclei in blue. White arrows indicate the location of bacteria, and scale bars are sown in the images. (C) Neutrophil signal area within a 50 µm radius of infected (red circles) or random non-infected (blue circles) macrophages, at 30 minutes and 5h post-infection of the porcine spleen. Data are representative of entire tissue sections (~2cm^2) from 3 replicate organs. Statistical significance was determined using a 1-way ANOVA. ***; $p<0.0005$, **; $p<0.005$, *, NS; $p>0.05$. (D) Neutrophil influx to the entire liver tissue section (30 min black, 5h red). Whole tissue sections from 3 replicate organs were analysed. Data are expressed as neutrophil fluorescence signal area per unit whole tissue area. (E) Representative confocal microscopy images of 30 minute and 5h infected porcine livers and spleens. Bacteria are shown in green, neutrophils are in magenta, and nuclei are shown in blue.

Supplementary Methods

Bacterial strains and culture conditions

Thirteen *Kp* strains of clinical origin, encompassing 5 capsular serotypes, including three hv*Kp* strains of serotype K1, three of K2, three non-hvKp carbapenem-resistant (CR) strains of serotype KL17, one of serotype KL103, and three of KL107, were used (Table 1; for full set of strain metadata see Table S1. The hypermucoid phenotype of all strains was confirmed by the string-test method [9]. Strains of *Kp* were growth in Brain Heart Infusion (BHI, Oxoid) broth for liquid culture, and on Lysogeny Broth Agar (LA, Oxoid) as a solid medium, at 37ºC. All bacteria were stored at -80ºC, in BHI broth supplemented with 10% v/v glycerol (Sigma). For infection stocks, bacteria were incubated overnight and then diluted 1:100 into fresh BHI and cultured to an optical density (OD_{600nm}) of 0.3. Samples were frozen at -80ºC, with 10% glycerol and one aliquot was counted prior to infection.

Bacteria growth assays

For assessing bacterial growth, colonies were suspended in PBS to an OD_{600nm} of 0.2, and then diluted 1:100 into either BHI medium, whole blood, or serum. Cultures were incubated in flat-bottomed 96-well microtiter plates (Sigma) in a plate reader at 37ºC, and OD_{600nm} values monitored every 5 minutes for 24h in the case of BHI, whereas blood and serum cultures were analysed by serial dilution and colony counting.

Rabbit IgG purification and fluorophore conjugation

IgG was purified from the rabbit K1 or K2 sera (Statens Serum Institut, Copenhagen) using a protein-G agarose kit (Roche). The protein concentration of the IgG preparation was determined by the Bradford assay, using bovine serum albumin as a standard. Purified anti-K1 and K2 IgG were conjugated with Texas red, and FITC respectively, using the easy FITC/Texas red labelling kits (Abcam), according to the manufacturer’s instructions.

Confocal microscopy, whole tissue section scanning microscopy, and image analysis

For analysis of bacterial distribution in tissues, immunostained tissue sections were imaged on a Vectra Polaris digital pathology system (Perkin Elmer; hereafter referred to as a ‘slide scanner’) using a 40x/NA=0.75 objective and Opal480, 520, 620, and 690 filter cubes. For quantitative analysis of the co-localisation of bacteria with particular cell populations in Vectra Polaris slide scans, an analysis pipeline was developed in Fiji (Figure S1). To delineate subcellular localisation of bacteria, Z-stacks of tissue samples stained with macrophage surface markers were acquired with an FV1000 Olympus Confocal laser scanning microscope

using a 60x/NA=1.35 objective and consistent step sizes of 0.25 µm. Confocal microscopy images were visualised in Fiji and Imaris 3D V9.4 reconstruction software was used for 3D visualisation of Z-stacks (Bitplane, Switzerland). Line scan analysis of confocal images was performed using a custom macro written in Fiji. For light microscopy imaging of haematoxylin and eosin stains, a fully motorised Nikon eclipse Ti microscope with a Nikon DS-Fi2 colour camera and a Plan Apo TIRF 100X oil objective was used. For scanning microscopy analysis, at least 2 tissue sections were analysed per mouse, whereas for confocal analysis, we display images representative of greater than 10 fields of view. In all cases, control tissue sections with the addition of no primary antibodies were performed.

Murine neutrophil isolation and bactericidal assay

Blood was collected from CD1 mice by cardiac puncture, under terminal anaesthesia, into EDTA vacutainers (BD Diagnostics-Preanalytical Systems, UK). Neutrophils were isolated by gradient centrifugation, as before [10], using Histopaque-1077 (Sigma, UK) and Histopaque-1119 (Sigma-Aldrich, UK). Neutrophil purity was assessed by confocal microscopy with anti-Ly6G antibody (Table S1). For bactericidal assays, frozen stocks of *Kp* were suspended in HBSS with Ca^{2+}/Mg^{2+} before infecting the neutrophil suspension at a MOI of 10 and incubation for 2h at 37ºC under rotation.

Culture and infection of J774a murine macrophages

J774.A1 mouse ascites macrophages (ATCC® TIB-67™) were grown in RPMI (Thermo Fisher, US) + 10% FBS and routinely passaged at 80% confluency. 96-well plates (Thermo Fisher, US) and 8-well chamber slides (Thermo Fisher, US) were seeded at a density of $1x10^5$ cells/ml and incubated overnight.

Overnight cultures of *K. pneumoniae* strains were diluted 1:100 and grown to an OD_{600} of 0.4. 30 minutes before infection. *K. pneumoniae* was added to the cells at an MOI of 10, and the plates were centrifuged at 200 x g for 5 minutes to synchronise infection of the cells. Following 30 minutes incubation at 37ºC, 5% CO_2, bactericidal antibiotics (300µg/ml Gentamicin + 15µg/ml Polymyxin B) were added for a further 30 minutes. The 4-hour post-infection wells were replaced with antibiotic at the minimum inhibitory concentration for each strain. At each time point, cells were lysed with 0.1% w/v Saponin, serially diluted 10-fold in BHI and plated for CFU enumeration. Chamber slide wells were fixed and stained for immunofluorescence as before [12].

***Ex vivo* gentamicin protection assay**

Four 6-8 weeks old female CD1 mice were intravenously infected with 10^6 CFU *Kp* strain NTUH-k2044 into the lateral tail vein. At 4 hours post-infection, mice were culled as per the

home office license for retrieval of livers, which were kept separate throughout the entirety of the experiment. Each liver was subject to digestion via injection with enzymatic cocktail solution, consisting of 32mg/ml Collagenase D (Sigma, UK) and 0.1% w/v DNase (Sigma, UK), and incubation at 37°C for 30 minutes. Livers were then gently homogenised through a 100µm cell strainer (Sigma, UK), and erythrocytes were lysed by addition of Gey's lysis solution whilst on ice for 3 minutes. Cells were washed thrice for removal of cell debris, before hepatocytes were pelleted via a 50 x g centrifugation for 3 minutes at 4°C. The supernatant was collected, and cell density and viability were determined. Three lots of $1x10^6$ cells were subject to either i) 37°C incubation for 1 hour with bactericidal antibiotics (DMEM (Thermo Fisher, US) + 10% FBS (Sigma, UK) + 300µg/ml Gentamicin + 15µg/ml Polymyxin B), three washes in PBS then lysis by addition of 0.1% w/v Saponin (Sigma, UK) ii) Lysis by 0.1% w/v Saponin, 5000 x g centrifugation for 10 minutes to pellet bacteria, resuspension in bactericidal antibiotic solution as above, and three washes in PBS or iii) Lysis by 0.1% w/v Saponin only. In all instances, the final solution was 10-fold serially diluted in BHI and plated for CFU enumeration.

Whole cell ELISA

K2 strain GMR151 was streaked onto LA plates, and incubated overnight at 37°C. The following day, single colonies were inoculated into 5mL of LB broth, and incubated o/n at 37 °C. The following day, samples were sub-cultured 1:100 into fresh LB broth and grown at 37 °C until an OD_{600} of 0.4. Samples were then pelleted at 3000 X G for 10 minutes, washed 3x in PBS, and resuspended in dH_2O to a final OD_{600nm} of 0.4. 100µl of bacterial suspension was dispensed into wells of the ELISA plate, which were dried o/n at room temperature in a biological safety cabinet to coat. The following day, samples were fixed in 100 µl of methanol, and left to dry o/n in a BSc. The following day, un-coated bacteria were removed, wells were washed 3x with PBS before proceeding with the ELISA protocol.

All steps of the ELISA protocol were performed at room temperature. Coated wells were first washed 3x with PBS supplemented with 0.05% (v/v) tween20 (Sigma). Wells were then incubated for 1h with 100µl of PBS supplemented with 5% (w/v) skimmed milk powder (Oxoid), 0.05% (v/v) tween20, and 0.001% (v/v) naïve goat serum (Sigma), to block non-specific binding sites. Samples were then probed for 1h with 50 µl of the sera of interest, diluted 2-fold in blocking buffer starting from 1:8, to a maximum dilution of 1:16384, including a no sera negative control. Plates were then washed 3x in wash buffer and probed for 1h with a secondary antibody conjugated

with horseradish peroxidase raised against either murine (Sigma), or porcine (Abcam) IgG (diluted 1:40,000 and 5000 respectively). Samples were then washed 3x in wash buffer and incubated for 10 min with 50 μl of tetramethylbenzidine (TMB; Sigma). The reaction was then stopped by adding 50 μl of 1M sulphuric acid (Sigma), before measuring the absorbance at 450nm using an Eon (Biotek) plate reader. All ELISAs were performed in at least duplicate.

Supplementary references

1. Fang CT, Chuang YP, Shun CT, Chang SC, Wang JT. A Novel Virulence Gene in Klebsiella pneumoniae Strains Causing Primary Liver Abscess and Septic Metastatic Complications. *Journal of Experimental Medicine*. 2004; 199(5):697-705.
2. Arena F, Spanu T, de Angelis LH, et al. First case of bacteremic liver abscess caused by an ST260-related (ST1861), hypervirulent Klebsiella pneumoniae. *Journal of Infection*. 2016; 73(1):88-91.
3. Lee IR, Molton JS, Wyres KL, et al. Differential host susceptibility and bacterial virulence factors driving Klebsiella liver abscess in an ethnically diverse population. *Scientific Reports*. 2016; 6:29316.
4. Cannatelli A, D’Andrea MM, Giani T, et al. In vivo emergence of colistin resistance in Klebsiella pneumoniae producing KPC-type carbapenemases mediated by insertional inactivation of the PhoQ/PhoP mgrB regulator. *Antimicrobial Agents and Chemotherapy*. 2013;57(11):5521-5526.
5. Castronovo G, Clemente AM, Antonelli A, et al. Differences in inflammatory response induced by two representatives of clades of the pandemic st258 Klebsiella pneumoniae clonal lineage producing KPC-type carbapenemases. *PLoS ONE*. 2017; 12(6):e0178847.
6. Bi D, Jiang X, Sheng ZK, et al. Mapping the resistance-associated mobilome of a carbapenem-resistant Klebsiella pneumoniae strain reveals insights into factors shaping these regions and facilitates generation of a “resistance-disarmed” model organism. *Journal of Antimicrobial Chemotherapy*. 2015;70(10):2770-2774.
7. Arena F, di Pilato V, Vannetti F, et al. Population structure of KPC carbapenemase-producing Klebsiella pneumoniae in a long-term acute-care rehabilitation facility: Identification of a new lineage of clonal group 101, associated with local hyperendemicity. *Microbial Genomics*. 2020;6(1):e000308..
8. Lee HC, Chuang YC, Yu WL, et al. Clinical implications of hypermucoviscosity phenotype in Klebsiella pneumoniae isolates: Association with invasive syndrome in patients with community-acquired bacteraemia. *Journal of Internal Medicine*. 2006;259(6):606-614.
9. Muntaka S, Almuhanna Y, Jackson D, et al. Gamma interferon and interleukin-17A differentially influence the response of human macrophages and neutrophils to pseudomonas aeruginosa infection. *Infection and Immunity*. 2019;87(2):e00814-18.
